# Post-traumatic stress disorder, anxiety, depression and burnout in nursing home staff in South France during the COVID-19 pandemic

**DOI:** 10.1038/s41398-023-02488-1

**Published:** 2023-06-15

**Authors:** Ismael Conejero, Melissa Petrier, Pascale Fabbro Peray, Christelle Voisin, Philippe Courtet, Hugo Potier, Loubna Elotmani, Brigitte Lafont, Jean-Yves Lefrant, Jorge Lopez Castroman, Christophe Arbus, Hubert Blain

**Affiliations:** 1 Department of Psychiatry, CHU Nîmes, PSNREC, INSERM, University of Montpellier, Nîmes, France; 2 Department of Biostatistics, Epidemiology, Public Health and Innovation in Methodology (BESPIM), CHU Nimes, IDESP, INSERM, University of Montpellier, Nîmes, France; 3 Agence Régionale de Santé Occitanie, Services régionaux de Toulouse, Direction de l’offre de soins et de l’autonomie, Pôle médico-social, Unité politique du vieillissement, Toulouse, France; 4 PSNREC, Univ Montpellier, INSERM, CHU de Montpellier, Montpellier, France; 5 Department of Emergency Psychiatry and Acute Care, Lapeyronie Hospital, CHU Montpellier, Montpellier, France; 6 UR-UM103, IMAGINE, Department of Anesthesia Critical Care Emergency and Pain Medicine, CHU Nimes, University of Montpellier, Nîmes, France; 7 DRCI, CHU Nîmes, University of Montpellier, Nîmes, France; 8 Department of Psychiatry, Nimes University Hospital, Institut de Génomique Fonctionnelle, University of Montpellier, CNRS-INSERM, Montpellier, France; 9 Centro de Investigación Biomédica en Red de Salud Mental, Madrid, Spain; 10 Toulouse University Hospital, Toulouse, France; 11 Inserm U1214, University of Toulouse III, Toulouse, France; 12 Department of Internal Medicine and Geriatrics, University Hospital of Montpellier, Montpellier University, Centre Antonin Balmes, Montpellier, France

**Keywords:** Depression, Human behaviour

## Abstract

The high mortality rate in nursing homes during the COVID-19 pandemic may be linked to psychological disorders in staff. Hence, we assessed the prevalence and associated factors of probable post-traumatic stress disorder (PTSD), anxiety, depression, and burnout of nursing home staff during the COVID-19 pandemic in a cross-sectional study including 66 randomly selected nursing homes in southern France. 537 of the contacted 3 821 nursing home workers (14.0%) responded between April and October 2021. We collected information on center organization, severity of COVID-19 exposure, and socio-demographic information in an online survey. The prevalence of probable PTSD (PCL-5), anxiety and depressive disorders (Hospital Anxiety Depression Scale) and the sub-scores of burnout syndrome (Maslach Burnout Inventory Human Services Survey for Medical Personnel) were assessed. Probable PTSD was reported in 115/537 responders (21.4% (95% CI [18.0%–24.9%])). After adjustment, low-level exposure to COVID-19 in nursing home residents (AOR, 0.5; 95% CI [0.3–0.9]), fear of managing COVID-19 residents (AOR, 3.5; 95% CI [1.9–6.4]), conflicts with residents (AOR, 2.3; 95% CI, [1.2–4.4]), conflicts with colleagues (AOR, 3.6; 95% CI [1.7–8.6]), cancellation of leave (AOR, 4.8; 95% CI [2.0–11.7]) and temporary worker employment (AOR, 3.4; 95% CI [1.7–6.9]) were associated with higher prevalence of probable PTSD. The prevalence of probable anxiety and depression were 28.8% (95% CI [24.9%–32.7%]) and 10.4% (95% CI [7.8%–13.1%]), respectively. Psychological disorders were observed in nearly one third of nursing home workers during the COVID-19 pandemic. Hence, continuous surveys and preventive measures are needed in this particularly at-risk population.

## Introduction

Since December 2019, the coronavirus SARS-CoV-2 has caused a worldwide outbreak of respiratory illness COVID-19, ranging from asymptomatic to mild and severe progressive pneumonia, leading to death with an overall lethality of around 3% [1, 2]. Prior to vaccine availability, the therapeutic strategy was based on symptomatic treatments and social distancing [3, 4].

By November 2022, the COVID-19 pandemic had caused near 160 000 deaths in France [5]. Older patients were particularly at risk of death, leading to strict lockdown measures in nursing homes with visit limitations [6]. These restrictions and contaminations among residents led to conflicts between patients, staff members and visitors.

In the general population, quarantine, lockdown and isolation contributed to Post-Traumatic Stress Disorder (PTSD) syndrome in about 30% of the overall population [3]. In the hospital setting, a meta-analysis including 39,000 healthcare workers reported stress symptoms in 37% of participants, depression in 32% and sleep disturbances in 36% of respondents [7]. Another meta-analysis found that the prevalence of post-pandemic PTSD was 26.9% in healthcare workers [8].

Nursing homes workers are at particular risk of developing psychological consequences such as PTSD, anxiety disorders, depression, and burnout syndrome (BOS) according to studies performed in the first year of COVID-19 pandemic [9]. During the first COVID-19 outbreak (early 2020), Riello et al., reported a prevalence of moderate-to-severe anxiety and/or PTSD in 43% of 1071 nursing home workers in northern Italy [10]. Another study completed in the same period in Italy reported PTSD diagnosis in nearly 35% of nursing home workers [11]. A review of recent international literature highlighted that preexisting psychological distress and moral injury may have been exacerbated during the COVID-19 outbreak [12]. In France, only one cross sectional survey involving a small population of workers (N = 127) in six nursing homes including mostly medical staff has been published, reporting panic attacks in 22% of participants, followed by probable depression (17%), PTSD (10%), general anxiety disorder (9%), and substance use disorders (4%) [13]. No factors were associated with the presence of a probable mental disorder in this study, probably because of a lack of statistical power.

Such psychological consequences are likely to be due to: (1) the management of COVID-19 residents, with potential mortality; (2) the risk of infecting oneself or others; (3) conflicts with residents’ relatives, for instance as a consequence of visit restriction policies or following infection of residents [14]; (4) dealing with an unknown and potentially severe disease, as well as the vaccine hesitancy among residents and healthcare workers [15–18]; and (5) excessive workload and feelings of uncertainty.

Given the significant impact with long-term consequences for nursing home workers of the COVID-19 pandemic, studies evaluating psychological distress in the later phases of the pandemic are important in this population. Furthermore, research involving greater samples may help to identify specific factors related to probable psychiatric disorders in this population. This study aimed to assess the prevalence and associated factors of probable PTSD in workers of nursing homes in the region Occitanie, in Southern France. We also assessed signs of probable anxiety and depression, BOS and their related factors.

## Materials/subjects and methods

### Design

A multicenter cross-sectional nursing home-based online survey was carried out among nursing home workers in Occitanie, a region of 6 million inhabitants subdivided into 13 departments, in Southern France. This study was approved by the national ethics committee (N° Eudract 2020-A03541-38, CPP Sud-Est 2 ref SI: 21.02.08.73027), been carried out in accordance with the Declaration of Helsinki and registered on clinicaltrials.gov (NCT04916275) [19]. A free informed patient consent was obtained before each inclusion (information was given in the questionnaire and the first question of the questionnaire asked the responder to participate with the possibility to refuse). This report followed the Strengthening the Reporting of Observational Studies in Epidemiology (STROBE) reporting [20], and the Checklist for Reporting Results of Internet E-Surveys (CHERRIES). All nursing homes (*n* = 817) of the region were informed of the study and 100 nursing homes were randomly selected, according to department and COVID-19 status. Centers were classified into three categories according to their COVID-19 status listed by Santé Publique France database at study initiation: Institutions with at least one COVID-19 resident (COVID + ), with no COVID-19 resident (FREE-COVID), and unknown status (UNK). Nursing homes which refused to participate were replaced at random by one with the same COVID-19 status in the same department.

The survey included two questionnaires:

1. The center questionnaire focused on the organization of the nursing home:
  Different categories of the staff;
  Number of beds in 2020;
  Number of residents in 2019 and 2020 (who stayed at least one day each year);
  Type of institution (public, private for/not for profit);
  Adaptation of the nursing homes during the COVID-19 pandemic (increase in staff, educational program for the staff, psychological support, vaccination program);
  Number of residents infected by COVID-19 and number of deaths among COVID-19 residents.
2. The personnel self-questionnaire collected:
  Socio-demographic data during the pandemic;
  Professional characteristics (job title, experience), experience during the COVID-19 pandemic (emotions, family and professional relationships);
  Three validated questionnaires to screen for probable psychiatric disorders: the PTSD Checklist for DSM-5 (PCL-5) [21] to evaluate the presence of probable PTSD, the Hospital Anxiety and Depression Scale (HADS) [22, 23] to evaluate the presence of probable anxiety or depression, and the Maslach Burnout Inventory - Human Services Survey for Medical Personnel (MBI-HSS-MP) [24] to evaluate BOS.

### Study population

All staff of participating nursing homes, caregivers and non-caregivers, were eligible to participate. The inclusion criteria were: aged 18 and older, fluent French, and access to the internet. For completing the personal questionnaire, a site-specific web link and QR code were provided to members via the nursing home local investigator. The recruitment was initially planned from May to June 2021.

### Outcomes

The primary outcome was the presence of probable PTSD defined by PCL-5 score ≥32 [21].

The secondary outcomes were the presence of probable anxiety or depression, and BOS. Anxiety and depression were separately screened with HADS questionnaire for total score between 0 and 21, where a score of 0 to 7 indicated absence of disorder; 8 to 10 a doubtful disorder; and 11 to 21 a probable disorder [22]. BOS was assessed by the MBI-HSS-MP on 3 specific sub-scales: emotional exhaustion, depersonalization and personal accomplishment. The three sub-scores are presented separately.

### Nursing home COVID-19 exposure

COVID-19 exposure used the classification used by the French Agency “Direction de la Recherche des Études de l’Évaluation et des Statistiques” (DREES) in four categories: COVID-19 critical exposure: ≥ 10% or 10 COVID-19 resident deaths; COVID-19 severe exposure: ≥ 33% or 30 COVID-19 residents; unqualified episode: at least one COVID-19 resident; FREE-COVID: no resident with COVID-19 [25]. To constitute groups, we used only the two categories: FREE-COVID vs COVID + (3 other categories).

Nursing home data included: legal status; number and categories of worker; recruitment of temporary workers during the pandemic; and types of preventive measures (information, protection, vaccination, psychological support).

Individual data included responder characteristics: age, sex, changes in life-style during the pandemic (relationships with proxies, residence, addiction); and professional characteristics (personal job, and seniority in job/in the establishment).

All these factors were analyzed as potentially associated with probable PTSD, anxiety, depression, and BOS.

Questionnaires were tested by 20 independent caregivers not involved in nursing home care before the start of the study. Moreover these questionnaires could be completed from any web or mobile device, via the anonymous REDCap link [26].

### Statistical analysis

#### Sample size

Assuming a baseline prevalence of PTSD of 25% (FREE-COVID group) and an absolute increase of 10% in the COVID group, i.e. 35%, respecting the imbalance of the groups from the preliminary data (ratio COVID/FREE-COVID = 2.45), with a two-sided alpha risk of 5% and a power of 90%, the number of participants needed is 1075 [8, 27]. To achieve this objective and to implement a multivariable mixed model with a clustering effect, we planned to include approximately 100 nursing homes, potentially representing 3750 staff members.

#### Statistical analysis

PTSD was classified as “probable” (PCL-5 score ≥ 32) versus no PTSD (PCL-5 score < 32) and presented with 95% confidence interval (95% CI) [21]. The prevalence of PTSD was estimated in the total sample and in the FREE-COVID and COVID+ groups.

PTSD associated factors were analyzed in a univariate analysis. The qualitative variables (expressed as numbers and percentages) were compared according to the PTSD by the chi-square test or the Fisher exact test as appropriate. The links between the explanatory variables and PTSD variable were expressed by odds ratios and 95% CI by the Wald method. Covariates with a *p*-value ≤ 0.20 in the univariate analysis were selected for multivariate analysis. To account for the hierarchical nature of the data (individual nested within the nursing home), the nursing home was defined as a random factor and included in a multilevel logistic regression with backward selection strategy at the 5% threshold. As main exposure, nursing-home COVID status was forced in the model. Adjusted odds ratio (AOR) were provided with 95% CI.

The same analysis strategy was applied to evaluate the prevalence of anxiety and depression and their associated factors. A multilevel polytomous logistic regression with proportional-odds cumulative logit model was used to search for factors associated with anxiety and depression classified in a three-level ordinal variable. The scores of the emotional exhaustion, depersonalization and personal accomplishment subscales were expressed as mean, standard deviation, median and interquartile range (IQR). The associated factors to the three sub-scores were assessed with a multilevel multiple linear regression model. The same variable selection strategy was used as for the previous models. Pearson correlation coefficients between PTSD, anxiety, depression, emotional exhaustion, depersonalization and personal accomplishment scores are provided with their 95% CI. All statistical analyses used SAS statistical software, version 9.4 (SAS Institute Inc).

## Results

**Fig. 1:**
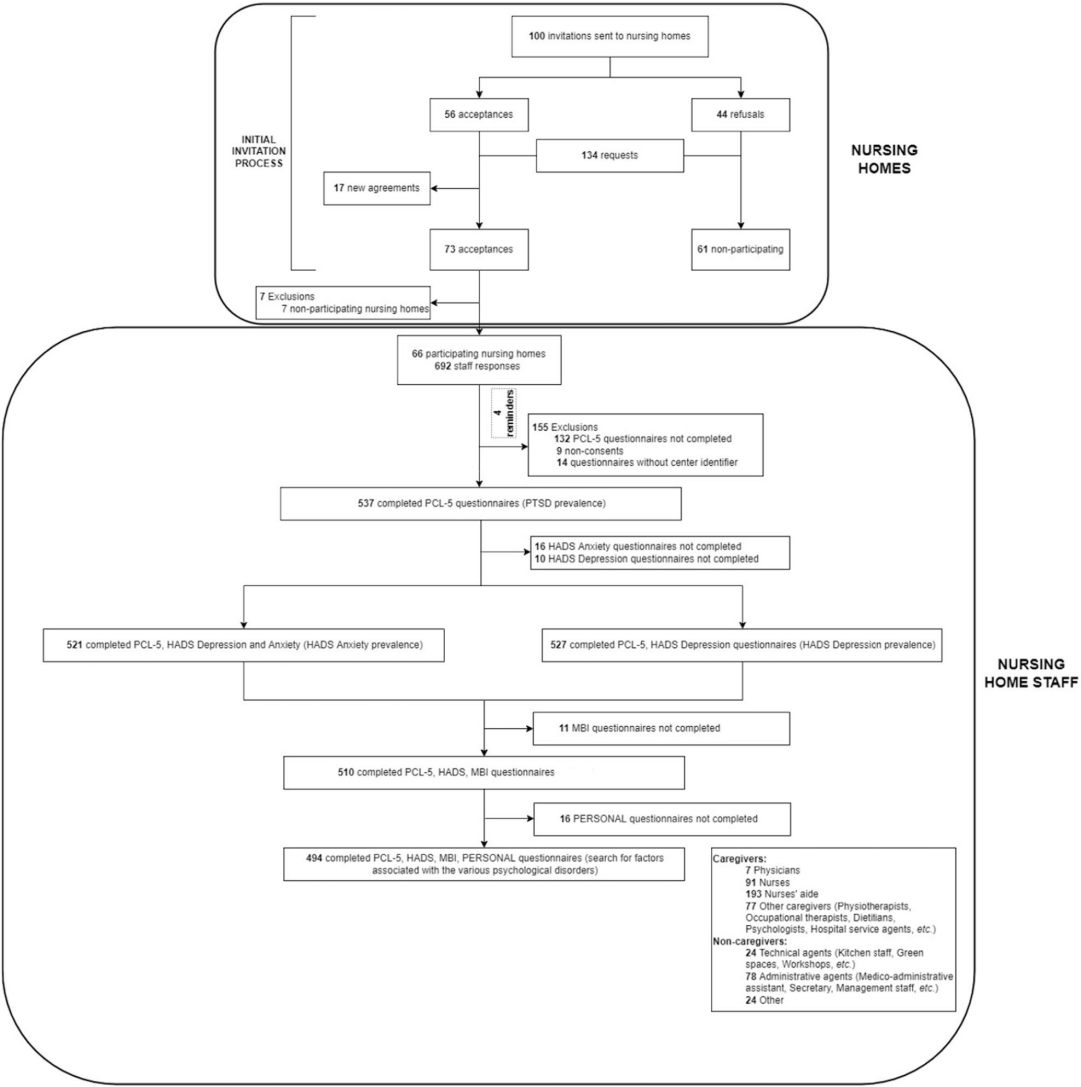
Flowchart illustrating the selection and participation to the study. PCL-5 PTSD Checklist for DSM-5, PTSD Post-Traumatic Stress Disorder, HADS Hospital Anxiety and Depression Scale, MBI Maslach Burnout Inventory.

Of the 817 eligible nursing homes, 134 were invited to participate and 66 agreed to respond (Fig. 1). Participating centers according to location and type of institution are shown in Fig. 1 and 2 of Supplemental Material. Among the 3821 potential workers, 537 (14%) responded to the PCL-5 questionnaire and 494 (13%) completed all questionnaires between 1st April and 11th October 2021. Among these 494 subjects, 368 (74.5%) were caregivers (193 nurses aids (39.1%), 91 nurses (18.4%), 7 physicians (1.4%), and 77 ‘others’ (15.6%)) and 126 (25.5%) were non-caregivers (78 administrative agents (15.8%), 24 technical agents (4.9%), and 24 ‘others’ (4.8%)).

Participants completed the various questionnaires at similar levels: PTSD (*n* = 537), anxiety (*n* = 521) and depression (*n* = 527) and burnout (*n* = 510) (Fig. 1). Response rate varied from 1% to 83% between the nursing homes (Figure 3 of Supplemental Material).

### PTSD

A PCL-5 score ≥ 32 was reported in 115/537 responders (21.4% 95% CI [18.0%–24.9%]) with no difference between responders from FREE-COVID (prevalence = 28/129, 21.7% 95% CI [14.6%–28.8%]) and COVID+ (prevalence = 87/408, 21.3% 95% CI [17.4%–25.3%]) nursing homes.

The multivariate analysis identified five factors associated with greater frequency of probable PTSD (Table 1, and Table 1 of Supplemental material): four worker characteristics (fear of managing COVID-19 residents, conflicts with residents, conflicts with colleagues, negative reaction to canceled leave) and one nursing home characteristic (temporary worker employment before the pandemic). There was no association between probable PTSD and working status (i.e., caregivers vs non-caregivers). High exposure to COVID-19 in nursing home residents was associated with a lower prevalence of PTSD.

**Table 1:** Independent factors associated with the presence of probable PTSD after multivariate analysis.

*N* = 494^*^	Probable PTSD, No^ƚ^. /Total No. (%)	Univariate analysis^ǂ^		Multivariate analysis^*^ (*N* = 472)	
		OR (95% CI)	*p*-value	AOR (95% CI)^§^	*p*-value
**Staff characteristics**					
**Fear for managing COVID-19 residents**					
No/Somewhat	43/293 (14.7)	1 [Reference]	**<0.0001**	1 [Reference]	**0.0002**
Moderately	22/96 (22.9)	1.8 (1.0–3.1)		2.1 (1.1–3.9)	
A lot	41/99 (41.4)	4.1 (2.4–6.9)		3.5 (1.9–6.4)	
Missing Data	6 (1.2)				
**Conflicts with colleagues**					
Never/rarely/sometimes	81/448 (18.1)	1 [Reference]	**<0.0001**	1 [Reference]	**0.001**
Frequently/very frequently	23/40 (57.5)	5.9 (3.0–11.8)		3.9 (1.6–9.0)	
Missing data	6 (1.2)				
**Conflicts with residents**					
Never/rarely/sometimes	74/416 (17.8)	1 [Reference]	**<0.0001**	1 [Reference]	**0.008**
Frequently/very frequently	31/73 (42.5)	3.4 (2.0–5.9)		2.3 (1.2–4.4)	
Missing data	5 (1.0)				
**Reaction to holiday cancellation**					
- Not concerned/It’s normal, I’m there for that, I’m useful to the residents/It’s part of the job, but it’s difficult, I’m tired	74/408 (18.1)	1 [Reference]	**<0.0001**	1 [Reference]	**0.002**
- This is part of the job but I think that there are not enough of us and it is always the same people who work more	11/47 (23.4)	1.4 (0.7–2.8)		0.9 (0.4–2.1)	
- I’m so tired that I’m thinking of changing my assignment or job	18/28 (64.3)	8.1 (3.6–18.3)		4.8 (2.0–11.7)	
Missing data	11 (2.2)				
**Nursing homes characteristics**					
**Level of severity of the episode**					
FREE-COVID/unqualified episode	74/312 (23.7)	1 [Reference]	0.13	1 [Reference]	**0.03**
Severe episode/critical episode	32/178 (18.0)	0.7 (0.4–1.1)		0.5 (0.3–0.9)	
Missing data	4 (0.8)				
**Use of temporary workers before the pandemic**					
No	22/167 (13.2)	1 [Reference]	**0.001**	1 [Reference]	**0.002**
Sometimes	52/218 (23.9)	2.1 (1.2–3.6)		1.6 (0.9–3.0)	
Often	32/98 (32.7)	3.2 (1.7–5.9)		3.4 (1.7–6.9)	
Missing data	11 (2.2)				

This table shows only independent factors significantly associated with the presence of probable PTSD after multivariate analysis.

Other variables included in the initial model but not significantly associated with the presence of probable PTSD are listed below:

- Age (<30 and ≥50 versus 30–49).

- Housing type (apartment versus house).

- Possession of an exterior to the dwelling (No versus yes).

- Frequency of information during the pandemic compared to before (less often versus with the same frequency versus more often).

- Feelings about the communication of COVID-19 pandemic medical data by the media (medium or total confidence versus distrust and low confidence).

- Feelings on media communication about personal preventing measures (medium or total confidence versus distrust and low confidence).

- Feelings on media communication about the means to fight measures (medium or total confidence versus distrust and low confidence).

- Perception of information about the pandemic in the center (insufficient versus medium versus excellent).

- Perception of the level of personal protective equipment (insufficient versus medium versus sufficient).

- Personal shortage (never/rarely/sometimes versus frequently/very frequently).

- Conflicts with resident relatives (never/rarely/sometimes versus frequently/very frequently).

- Accompanying COVID residents at the end-of-life (No versus Yes).

- People at risk for severe COVID-19 (No versus Yes).

- Transfer of COVID residents to another institution (No versus Yes).

^*^According to the order of appearance of the survey forms, an imbalance in the completion rate was noted between the first questionnaire (PCL-5) and the last form (Socio-demographic questionnaire used to research the factors associated with the psychological disorders studied) (higher completion rate for the first questionnaire). In order to evaluate the prevalence associated with psychological disorders, all the answers filled in for each scale of evaluation of the latter were taken into account, although the questionnaire was not completed in full. For this reason, a difference in the numbers analyzed (between those for the prevalence of post-traumatic stress and those for the analysis of associated factors) is observed (see Fig. 1). The search for factors associated with the occurrence of psychological disorders was carried out on 494 people (those who completed all the survey forms).

*PTSD* Post-Traumatic Stress Disorder.

^ƚ^No./Total No.: Number of observations/Total number of observations.

^ǂ^The results presented correspond to the pre-selection of variables at *p*-value ≤ 20%. A second selection of variables was made at the 5% threshold and then integrated into the multivariate model.

^**§**^AOR: Adjusted odd ratio for all the variables of the table with 95% confidence interval.

PCL-5 score was highly correlated with anxiety (*r* = 0.72, 95% CI [0.67–0.75], *p* < 0.0001), depression (*r* = 0.64, 95% CI [0.59–0.69], *p* < 0.0001) and emotional exhaustion (*r* = 0.62, 95% CI [0.56–0.67], *p* <0 .0001) scores (Table 2 of Supplemental Material).

### Anxiety

A probable anxiety disorder was reported in 150/521 responders (28.8%, 95% CI [24.9%–32.7%]) with no difference between responders from FREE-COVID (prevalence = 41/127, 32.3% 95% CI [24.2%–40.4%]) and COVID+ (prevalence = 109/394, 27.7% 95% CI [23.3%–32.1%]) nursing homes. According to the multivariate analysis, fear of managing COVID-19 residents, conflicts with residents, age between 30 and 49 year-old, family environment alteration and distrust or low confidence in the data relayed by the media, were associated with anxiety disorder (Table 2, and Table 3 of Supplemental material). There was no association between probable anxiety and working status.

**Table 2:** Independent factors associated with the anxiety gradient after multivariate analysis.

ANXIETY					
*N* = 494^*^	No^ƚ^. /Total No. (%)	Univariate analysis^ǂ^		Multivariate analysis^*^ (*N* = 479)	
		OR (95% CI)	*p*-value	AOR (95% CI)^§^	*p*-value
**Staff characteristics**					
**Fear for managing COVID-19 residents**					
No/Somewhat	62/290 (21.4)	1 [Reference]	**<0.0001**	1 [Reference]	**<0.0001**
Moderately	31/94 (33.0)	1.9 (1.2–3.0)		1.8 (1.1–2.8)	
A lot	47/99 (47.5)	3.2 (2.0–4.9)		2.8 (1.7–4.4)	
Missing Data	11 (2.2)				
**Age (years)**					
<30 and ≥50	61/245 (24.9)	1 [Reference]	**0.01**	1 [Reference]	**0.002**
30–49	79/239 (33.1)	1.5 (1.1–2.2)		1.7 (1.2–2.5)	
Missing Data	10 (2.0)				
**Feelings about the communication of COVID-19 pandemic medical data by the media**					
Medium or total confidence	58/250 (23.2)	1 [Reference]	**0.0001**	1 [Reference]	**0.0002**
Distrust or low confidence	80/233 (34.3)	2.0 (1.4–2.8)		2.0 (1.4–2.8)	
Missing data	11 (2.2)				
**Same family environment as before the pandemic**					
No	12/32 (37.5)	1 [Reference]	**0.04**	1 [Reference]	**0.009**
Yes	128/453 (28.3)	0.5 (0.2–0.9)		0.4 (0.2–0.8)	
Missing Data	9 (1.8)				
**Conflicts with residents**					
Never/Rarely/Sometimes	102/412 (24.8)	1 [Reference]	**<0.0001**	1 [Reference]	**<0.0001**
Frequently/Very frequently	37/72 (51.4)	3.3 (2.0–5.4)		2.9 (1.8–4.9)	
Missing Data	10 (2.0)				

This table shows only independent factors significantly associated with the anxiety gradient after multivariate analysis.

Other variables included in the initial model but not significantly associated with the anxiety gradient are listed below:

- Personal shortage (never /rarely/sometimes versus frequently/very frequently).

- Feelings on media communication about personal preventing measures (medium or total confidence versus distrust or low confidence).

- Feelings on media communication about the means to fight measures (medium or total confidence versus distrust or low confidence).

- Perception of information about the pandemic in the center (insufficient versus medium versus excellent).

- Perception of the level of personal protective equipment (insufficient versus medium versus sufficient).

- Conflicts with colleagues (never/rarely/sometimes versus frequently/very frequently).

- Level of severity of the episode (free-covid/unqualified episode versus severe episode/critical episode).

^*^According to the order of appearance of the survey forms, an imbalance in the completion rate was noted between the first questionnaire (PCL-5) and the last form (Socio-demographic questionnaire used to research the factors associated with the psychological disorders studied) (higher completion rate for the first questionnaire). In order to evaluate the prevalence associated with psychological disorders, all the answers filled in for each scale of evaluation of the latter were taken into account, although the questionnaire was not completed in full. For this reason, a difference in the numbers analyzed (between those for the prevalence of anxiety and those for the analysis of associated factors) is observed (see Fig. 1). The search for factors associated with the occurrence of psychological disorders was carried out on 494 people (those who completed all the survey forms).

^ƚ^No./Total No.: Number of observations/Total number of observations.

^ǂ^The results presented correspond to the pre-selection of variables at *p*-value ≤ 20%. A second selection of variables was made at the 5% threshold and then integrated into the multivariate model.

^**§**^AOR: Adjusted odd ratio for nursing-home COVID status (FREE-COVID/Unqualified episode versus Severe episode/Critical episode) and all the variables of the table with 95% confidence interval.

### Depression

A probable depressive disorder was reported in 55/527 responders (10.4% 95% CI [7.8%–13.1%]) with no difference between responders from FREE-COVID (prevalence = 20/131, 15.3% 95% CI [9.1%–21.4%]) and COVID+ (prevalence = 35/396, 8.8% 95% CI [6.0%–11.6%]) nursing homes.

According to the multivariate analysis, comorbidity at risk of severe COVID-19, distrust or low confidence in media communication about personal preventive measures, conflicts with residents and colleagues, negative reaction to canceled leave and insufficient information provided by the institution, were associated with depressive disorder (Table 3, and Table 4 of Supplemental material). There was no association between probable depression and working status.

**Table 3:** Independent factors associated with the depression gradient after multivariate analysis.

DEPRESSION					
*N* = 494^*^	No^ƚ^. /Total No. (%)	Univariate analysis^ǂ^		Multivariate analysis^*^ (*N* = 476)	
		OR (95% CI)	*p*-value	AOR (95% CI)^§^	*p*-value
**Staff characteristics**					
**People at risk of developing a severe form of COVID-19**					
No	32/370 (75.5)	1 [Reference]	**0.001**	1 [Reference]	**0.003**
Yes	18/120 (15.0)	2.0 (1.3–3.1)		2.0 (1.3–3.2)	
Missing data	4 (0.8)				
**Feelings on media communication about personal preventing measures**					
Medium or total confidence	31/337 (9.2)	1 [Reference]	**0.006**	1 [Reference]	**0.01**
Distrust or low confidence	19/150 (12.7)	1.8 (1.2–2.7)		1.8 (1.1–2.7)	
Missing Data	7 (1.4)				
**Conflicts with colleagues**					
Never/rarely/sometimes	34/449 (7.6)	1 [Reference]	**<0.0001**	1 [Reference]	**0.02**
Frequently/very frequently	15/39 (38.5)	4.0 (2.1–7.7)		2.3 (1.1–4.7)	
Missing data	6 (1.2)				
**Conflicts with residents**					
Never/rarely/sometimes	37/417 (8.9)	1 [Reference]	**0.0002**	1 [Reference]	**0.003**
Frequently/very frequently	13/72 (18.1)	2.7 (1.6–4.5)		2.3 (1.3–4.0)	
Missing data	5 (1.0)				
**Reaction to vacation cancellation**					
- Not concerned/It’s normal, I’m there for that, I’m useful to the residents/It’s part of the job, but it’s difficult, I’m tired	34/408 (8.3)	1 [Reference]	**<0.0001**	1 [Reference]	**0.001**
- This is part of the job but I think that there are not enough of us and it is always the same people who work more	4/47 (8.5)	1.2 (0.6–2.4)		(0.5–2.1)	
- I’m so tired that I’m thinking of changing my assignment or job	12/29 (41.4)	5.6 (2.7–11.8)		4.1 (1.9–8.8)	
Missing data	10 (2.0)				
**Perception of information about the pandemic in the center**					
Insufficient	16/54 (29.6)	1 [Reference]	**<0.0001**	1 [Reference]	**0.01**
Medium	12/169 (7.1)	0.3 (0.2–0.6)		0.5 (0.3–1.0)	
Excellent	22/264 (8.3)	0.3 (0.1–0.5)		0.4 (0.2–0.7)	
Missing data	7 (1.4)				

This table shows only independent factors significantly associated with the depression gradient after multivariate analysis.

Other variables included in the initial model but not significantly associated with the depression gradient are listed below:

- Fear for managing COVID-19 residents (No/Somewhat versus moderately versus a lot).

- Feelings about the communication of COVID-19 pandemic medical data by the media (medium or total confidence versus distrust or low confidence).

- Feelings on media communication about the means to fight measures (medium or total confidence versus distrust or low confidence).

- Conflicts with resident relatives (never/rarely/sometimes versus frequently/very frequently).

- Level of severity of the episode (FREE-COVID/Unqualified episode versus Severe episode/Critical episode).

- Transfer of COVID residents to another institution (No versus yes).

^*^According to the order of appearance of the survey forms, an imbalance in the completion rate was noted between the first questionnaire (PCL-5) and the last form (Socio-demographic questionnaire used to research the factors associated with the psychological disorders studied) (higher completion rate for the first questionnaire). In order to evaluate the prevalence associated with psychological disorders, all the answers filled in for each scale of evaluation of the latter were taken into account, although the questionnaire was not completed in full. For this reason, a difference in the numbers analyzed (between those for the prevalence of depression and those for the analysis of associated factors) is observed (see Fig. 1). The search for factors associated with the occurrence of psychological disorders was carried out on 494 people (those who completed all the survey forms).

^ƚ^No./Total No.: Number of observations/Total number of observations.

^ǂ^The results presented correspond to the pre-selection of variables at *p*-value ≤ 20%. A second selection of variables was made at the 5% threshold and then integrated into the multivariate model.

^**§**^AOR: Adjusted odd ratio for nursing-home COVID status (FREE-COVID/Unqualified episode versus Severe episode/Critical episode) and all the variables of the table with 95% confidence interval.

### Burnout sub-scores

#### Emotional exhaustion

The mean score of emotional exhaustion in the total sample was 25.3 (±14.3). According to the multivariate analysis, fear of managing COVID-19 residents, conflicts with residents and colleagues, distrust or low confidence in the data relayed by the media, negative reaction to canceled leave and personnel shortage were independently associated with a greater emotional exhaustion. In contrast, the perception of excellent information from the institution decreased emotional exhaustion (Table 5 of supplemental material).

#### Depersonalization

The mean depersonalization score in the total sample was 7.3 (±6.5). According to the multivariate analysis, fear of managing COVID-19 residents, conflicts with residents and their relatives, distrust or low confidence in media communication about personal preventive measures, negative reaction to canceled leave and insufficient level of personal protective equipment were associated with a greater depersonalization. Nursing homes that used more temporary workers during the pandemic than before were associated with a greater depersonalization. Having transferred COVID-19 residents to another facility decreased depersonalization (Table 5 of supplemental material).

#### Personal accomplishment

The mean score of personal accomplishment in the total sample was 37.8 (±7.0). According to the multivariate analysis, fear of managing COVID-19 residents, conflicts with residents’ relatives and being a non-healthcare worker were associated with a lower personal accomplishment, while seeking information with the same frequency or more often during the pandemic than before was associated with greater personal accomplishment (Table 5 of supplemental material).

Emotional exhaustion and depersonalization scores were both highly correlated (*r* = 0.60, 95% CI [0.54–0.66], *p* < 0.0001). Depersonalization was negatively but less correlated with personal accomplishment (Table 2 of supplemental material). Data for each score are reported in Table 4.

**Table 4:** Dispersion and position parameters associated with the assessment scales for psychological disorders^*^.

Evaluation scale		N^**^	Missing data	Mean (SD)^ƚƚ^	Median (IQR)^ǂǂ^	Lower quartile	Upper quartile	Minimum	Maximum
**PCL-5 (Post Traumatic Stress Disorder)**		478	16	19.8 (15.2)	16.0 (22.0)	7.0	29.0	0	77.0
**HADS**	**Anxiety**	483	11	8.2 (3.9)	8.0 (6.0)	5.0	11.0	0	19.0
	**Depression**	485	9	5.4 (3.9)	5.0 (6.0)	2.0	8.0	0	19.0
**MBI**	**Emotional Exhaustion**	480	14	25.3 (14.3)	25.0 (24.0)	13.0	37.0	0	54.0
	**Depersonalization**	483	11	7.3 (6.5)	6.0 (9.0)	2.0	11.0	0	30.0
	**Personal Accomplishment**	469	25	37.8 (7.0)	39.0 (9.0)	34.0	43.0	0	48.0

*494 respondents completed PCL-5, HADS, MBI and Personal Questionnaires.

**N Number of participants who responded to all the questionnaire items.

^ƚƚ^*SD* Standard deviation.

^#^*IQR* Interquartile range, *HADS* Hospital Anxiety and Depression Scale, *MBI* Maslach Burnout Inventory, *PCL-5* PTSD Checklist for DSM-5.

## Discussion

Among 817 nursing homes, 66 of the 134 contacted participated to the study, with 13% global response rate. Probable PTSD was reported in 21.4% of responders with no difference between responders from FREE-COVID and COVID+ nursing homes. Fear of managing COVID-19 residents, conflicts with residents and colleagues, canceled leave and temporary worker employment were associated with a greater presence of PTSD, whereas a high level of exposure to COVID-19 was associated with a lower prevalence of PTSD. The prevalence of probable anxiety and depression were 28.8% and 10.4% respectively. The occurrence of conflicts with residents was associated with a greater prevalence of anxiety, depression, and a higher emotional exhaustion score. The occurrence of fear of managing COVID-19 residents was associated with a greater prevalence of PTSD, anxiety, and higher emotional exhaustion, depersonalization scores, and a lower personal accomplishment. The occurrence of canceled leave was associated with a greater prevalence of PTSD, depression, and higher emotional exhaustion and depersonalization scores.

The COVID-19 pandemic has numerous short- and long-term psychological consequences possibly arising from having been infected or from transmitting the virus to close family, preventive measures (for instance lockdown) and quarantining. Indeed, PTSD, anxiety, depression and burnout have been widely reported among the general population and in healthcare workers [28] and may be linked with increased rate of suicidal behavior [29, 30]. One month following the first lockdown in France, PTSD was reported in 14% of the general population using the PCL-5 with a cutoff score of 33 [31]. Our findings show higher rates of PTSD among nursing home workers, highlighting that nursing home workers are a particularly at-risk population. Another study conducted by the same team found moderate-to-severe anxiety symptoms in 21% of the general population, and moderate-to-severe depression symptoms in 23% of the population in the last 4 days of the first French lockdown [32]. These results are less comparable with our findings, due to different measure instruments used in this study (Patient Health Questionnaire-9 and the General Anxiety Disorder-7).

The restrictive visiting policies implemented in nursing homes exposed residents and employees (caregiver and administrative agents) to psychological disturbances [33]. In a qualitative assessment, Snyder et al., (2021) outlined that 68% of nursing home workers reported performing tasks beyond their scope of work and additional responsibilities of rule and protocol enforcement [34]. Also, the lack of systematic guidance and rapidly changing preventive protocols was reported as time consuming, and as a source of stress [34].

A cross-sectional survey of 390 nursing home staff performed during the third wave of COVID-19 pandemic in Ireland (from 20 November 2020 to 4 January 2021) found PTSD in 45.1% of all staff, low mood (38.7%, especially in nurses), and suicidal behavior (13.8% with no difference between job categories) [35]. Interestingly, moral injuries were more present in healthcare givers than in non-clinical staff. During the first COVID-19 wave in Northern Italy (from 15 June to 25 July 2020), Riello et al., reported moderate-to-severe anxiety and/or PTSD in 43% of 1071 nursing home workers [10]. In our study, PTSD prevalence was lower than in the Irish and Italian studies, possibly because of differences in COVID-19 incidence, the timing of the evaluation and specificities in cultural and social environment. Further studies should be conducted to investigate these specificities.

This study highlighted several factors associated with PTSD, anxiety, depression, and symptoms of BOS. The implication of conflicts with patients [36] and the cancellation of expected holidays leading to family conflicts [37] have already been related to psychological disorders among healthcare workers. Interestingly, temporary worker employment was associated with a greater risk of developing PTSD. Indeed, in their recent article, Ridley et al., underlined the association between economic uncertainty, income volatility and the risk of developing mental illnesses [38]. Hence, precarious economic status resulting from temporary work may increase the risk of PTSD among nursing home staff. This should be confirmed in further studies.

Although the fear of managing COVID-19 resident was associated with a higher prevalence of PTSD, a high level of exposure to COVID-19 in nursing home residents was associated with a lower one. This surprising finding could be explained by the degree of information and expertise in workers managing COVID-19 patients.

We must acknowledge certain limitations. First, participation rate was around 10%, as classically reported in cross-sectional survey without possibility of personal reminder (respecting responder anonymity). The personnel participation varied between institutions (1–83%). Only 66 of 134 contacted nursing homes agreed to participate. The timing of our study (after the third wave, June-July 2021) was perhaps too long after the start of the pandemic with participant weariness leading to a low response rate. Second, due to its cross-sectional survey design, our study could only determine associated factors with probable PTSD, anxiety, depression, and BOS, without any causal relationship. To isolate risk factors of these psychological disorders, cohort or case control designs might have been more appropriate, but none have been performed so far. Third, data on participant follow-up by mental health professionals and the intake of psychotropic medication at the time of the survey was not included in the analysis. Hence, prevalence of psychological disorder may have been underestimated. Yet, these factors were also not taken into account in other similar studies conducted in nursing homes [10, 35]. Fourth, while PCL-5 and HADS have been used in population survey studies [31, 39], these are screening instruments. The lack of clinical interview prevented us from confirming the diagnoses, that can only be considered as probable. The HADS was initially designed to detect depression and anxiety in hospitalized patients, however this has been effectively used in a wide variety of studies including non-admitted participants [40, 41]. Finally, private for-profit nursing homes were underrepresented.

This study shows that the COVID-19 pandemic was accompanied by a risk of developing psychological disorders in nearly a third of nursing home workers. Some factors linked to the COVID-19 pandemic (fear of managing COVID-19 patient), and others such as conflicts with residents and colleagues, and cancellation of leave were associated with a higher prevalence of probable PTSD, anxiety, depression and BOS. These findings could plea for continuing surveying nursing home workers, as this population is particularly at risk for psychological disorders. These surveys could lead to preventive and therapeutic measures.

## Supplementary information

Supplemental material (Tables)

Supplemental material (Figures)

## Data Availability

The data will be made available upon request.

## References

[1] Wilcox SR (2020). Management of respiratory failure due to covid-19. BMJ.

[2] Aslan A, Aslan C, Zolbanin NM, Jafari R (2021). Acute respiratory distress syndrome in COVID-19: possible mechanisms and therapeutic management. Pneumonia (Nathan).

[3] Brooks SK, Webster RK, Smith LE, Woodland L, Wessely S, Greenberg N (2020). The psychological impact of quarantine and how to reduce it: rapid review of the evidence. Lancet.

[4] Or Z, Gandré C, Durand Zaleski I, Steffen M (2022). France’s response to the Covid-19 pandemic: between a rock and a hard place. Health Econ Policy Law.

[5] WHO. COVID-19 Overview—Johns Hopkins. 2023. Available from. https://coronavirus.jhu.edu/region/france

[6] van Tol LS, Smaling HJA, Groothuijse JM, Doornebosch AJ, Janus SIM, Zuidema SU (2021). COVID-19 management in nursing homes by outbreak teams (MINUTES) - study description and data characteristics: a qualitative study. BMJ Open.

[7] Dutta A, Sharma A, Torres-Castro R, Pachori H, Mishra S (2021). Mental health outcomes among health-care workers dealing with COVID-19/severe acute respiratory syndrome coronavirus 2 pandemic: a systematic review and meta-analysis. Indian J Psychiatry.

[8] Yuan K, Gong YM, Liu L, Sun YK, Tian SS, Wang YJ (2021). Prevalence of posttraumatic stress disorder after infectious disease pandemics in the twenty-first century, including COVID-19: a meta-analysis and systematic review. Mol Psychiatry.

[9] Gray KL, Birtles H, Reichelt K, James IA (2022). The experiences of care home staff during the COVID-19 pandemic: a systematic review. Aging Ment Health.

[10] Riello M, Purgato M, Bove C, MacTaggart D, Rusconi E (2020). Prevalence of post-traumatic symptomatology and anxiety among residential nursing and care home workers following the first COVID-19 outbreak in Northern Italy. R Soc Open Sci.

[11] Faretta E, Maslovaric G, Garau MI, Marmondi G, Piras L, Rezzola S (2022). The psychological impact of the COVID emergency on Italian nursing homes staff and the effectiveness of eye movement desensitization and reprocessing. Front Psychol.

[12] Laher Z, Robertson N, Harrad-Hyde F, Jones CR (2022). Prevalence, predictors, and experience of moral suffering in nursing and care home staff during the COVID-19 pandemic: a mixed-methods systematic review. Int J Environ Res Public Health.

[13] Husky MM, Villeneuve R, Tabue Teguo M, Alonso J, Bruffaerts R, Swendsen J (2022). Nursing home workers’ mental health during the COVID-19 pandemic in France. J Am Med Dir Assoc.

[14] Freidus A, Shenk D (2020). « It Spread Like a Wildfire »: Analyzing affect in the narratives of nursing home staff during a COVID-19 outbreak. Anthropol Aging.

[15] Adhikari B, Cheah PY (2021). Vaccine hesitancy in the COVID-19 era. Lancet Infect Dis.

[16] Dzieciolowska S, Hamel D, Gadio S, Dionne M, Gagnon D, Robitaille L (2021). Covid-19 vaccine acceptance, hesitancy, and refusal among Canadian healthcare workers: A multicenter survey. Am J Infect Control.

[17] Shih SF, Wagner AL, Masters NB, Prosser LA, Lu Y, Zikmund-Fisher BJ (2021). Vaccine hesitancy and rejection of a vaccine for the Novel coronavirus in the United States. Front Immunol.

[18] Harrison J, Berry S, Mor V, Gifford D (2021). “Somebody like me”: understanding COVID-19 vaccine hesitancy among staff in skilled nursing facilities. J Am Med Dir Assoc.

[19] Toulouse E, Masseguin C, Lafont B, McGurk G, Harbonn A, A Roberts J (2018). French legal approach to clinical research. Anaesth Crit Care Pain Med.

[20] The Strengthening the Reporting of Observational Studies in Epidemiology (STROBE) Statement: guidelines for reporting observational studies | The EQUATOR Network. https://www.equator-network.org/reporting-guidelines/strobe/10.1136/bmj.39335.541782.AD PMC2034723 17947786

[21] Ashbaugh AR, Houle-Johnson S, Herbert C, El-Hage W, Brunet A (2016). Psychometric validation of the English and French versions of the posttraumatic stress disorder checklist for DSM-5 (PCL-5). PLoS One.

[22] Zigmond AS, Snaith RP (1983). The hospital anxiety and depression scale. Acta Psychiatr Scand.

[23] Lépine JP, Godchau M, Brun P (1985). Lempérière Th. Anxiety and depression evaluation in patients hospitalized in an internal medicine unit. Ann. Méd.-Psychol..

[24] Maslach C, Jackson S (1981). The measurement of experienced burnout. J Organ Behav.

[25] Accueil. Direction de la recherche, des études, de l’évaluation et des statistiques. 2022. https://drees.solidarites-sante.gouv.fr/publications/etudes-et-resultats/en-2020-trois-ehpad-sur-quatre-ont-eu-au-moins-un-resident-infecte

[26] Harris PA, Taylor R, Minor BL, Elliott V, Fernandez M, O’Neal L (2019). The REDCap consortium: Building an international community of software platform partners. J Biomed Inf.

[27] Cénat JM, Blais-Rochette C, Kokou-Kpolou CK, Noorishad PG, Mukunzi JN, McIntee SE (2021). Prevalence of symptoms of depression, anxiety, insomnia, posttraumatic stress disorder, and psychological distress among populations affected by the COVID-19 pandemic: a systematic review and meta-analysis. Psychiatry Res.

[28] Wathelet M, D’Hondt F, Bui E, Vaiva G, Fovet T (2021). Posttraumatic stress disorder in time of COVID‐19: trauma or not trauma, is that the question?. Acta Psychiatr Scand.

[29] Conejero I, Berrouiguet S, Ducasse D, Leboyer M, Jardon V, Olié E (2020). Épidémie de COVID-19 et prise en charge des conduites suicidaires: challenge et perspectives. Encephale.

[30] Conejero I, Nobile B, Olié E, Courtet P (2021). How does COVID-19 affect the neurobiology of suicide?. Curr Psychiatry Rep.

[31] Alleaume C, Peretti-Watel P, Beck F, Leger D, Vaiva G, Verger P (2022). Incidence of PTSD in the French population a month after the COVID-19 pandemic-related lockdown: evidence from a national longitudinal survey. BMC Public Health.

[32] Alleaume C, Verger P, Peretti-Watel P (2021). Group the C. Psychological support in general population during the COVID-19 lockdown in France: needs and access. PLOS ONE.

[33] White EM, Wetle TF, Reddy A, Baier RR (2021). Front-line nursing home staff experiences during the COVID-19 pandemic. J Am Med Dir Assoc.

[34] Snyder RL, Anderson LE, White KA, Tavitian S, Fike LV, Jones HN (2021). A qualitative assessment of factors affecting nursing home caregiving staff experiences during the COVID-19 pandemic. PLOS ONE.

[35] Brady C, Fenton C, Loughran O, Hayes B, Hennessy M, Higgins A, et al. Nursing home staff mental health during the Covid-19 pandemic in the Republic of Ireland. Int J Geriatr Psychiatry. 2021. 10.1002/gps.5648 10.1002/gps.5648 PMC8646737 34729818

[36] Anderson-Shaw LK, Zar FA (2020). COVID-19, moral conflict, distress, and dying alone. J Bioeth Inq.

[37] Frank E, Zhao Z, Fang Y, Rotenstein LS, Sen S, Guille C (2021). Experiences of work-family conflict and mental health symptoms by gender among physician parents during the COVID-19 pandemic. JAMA Netw Open.

[38] Ridley M, Rao G, Schilbach F, Patel V (2020). Poverty, depression, and anxiety: causal evidence and mechanisms. Science.

[39] Miniarikova E, Vernhet C, Peries M, Loubersac J, Picot MC, Munir K (2022). Anxiety and depression in parents of children with autism spectrum disorder during the first COVID-19 lockdown: report from the ELENA cohort. J Psychiatr Res.

[40] Silverberg JI, Gelfand JM, Margolis DJ, Boguniewicz M, Fonacier L, Grayson MH (2019). Symptoms and diagnosis of anxiety and depression in atopic dermatitis in U.S. adults. Br J Dermatol.

[41] Robb CE, de Jager CA, Ahmadi-Abhari S, Giannakopoulou P, Udeh-Momoh C, McKeand J (2020). Associations of social isolation with anxiety and depression during the early COVID-19 pandemic: a survey of older adults in London, UK. Front Psychiatry.

