# Supplementary material for: Post-traumatic stress disorder, anxiety, depression and burnout in nursing home staff in South France during the COVID-19 pandemic
Source: Transl Psychiatry. 2023 Jun 15;13:205. doi: 10.1038/s41398-023-02488-1 (PMC10267542; doi:10.1038/s41398-023-02488-1)
Supplement: Supplementary file 1 — Supplemental material (Tables) [file 41398_2023_2488_MOESM1_ESM.docx]

| **Table 1. Supplemental material. Factors associated with the presence of probable PTSD** | | | | | |
| --- | --- | --- | --- | --- | --- |
| **N = 494^*^** | **Probable PTSD,**  **No^ƚ^. /Total No. (%)** | **Univariate analysis^ǂ^** | | **Multivariate analysis^*^ (N = 472)** | |
|  |  | **OR (95% CI)** | **p value** | **AOR (95% CI)^§^** | **p value** |
| **Staff characteristics** |  |  |  |  |  |
| **Fear for managing COVID-19 residents** |  |  |  |  |  |
| No/Somewhat | 43/293 (14.7) | 1 [Reference] | **<.0001** | 1 [Reference] | **.0002** |
| Moderately | 22/96 (22.9) | 1.8 (1.0 – 3.1) |  | 2.1 (1.1 – 3.9) |  |
| A lot | 41/99 (41.4) | 4.1 (2.4 – 6.9) |  | 3.5 (1.9 – 6.4) |  |
| Missing Data | 6 (1.2) |  |  |  |  |
| **Age (years)** |  |  |  |  |  |
| < 30 and ≥ 50 | 47/249 (18.9) | 1 [Reference] | .12 | NA**^**^** | NA |
| 30 – 49 | 59/241 (24.5) | 1.4 (0.9 – 2.2) |  | NA | NA |
| Missing Data | 4 (0.8) |  |  |  |  |
| **Housing type** |  |  |  |  |  |
| Apartment | 27/100 (27.0) | 1 [Reference] | .18 | NA | NA |
| House | 79/389 (20.3) | 0.7 (0.4 – 1.2) |  | NA | NA |
| Missing Data | 5 (1.0) |  |  |  |  |
| **Possession of an exterior to the dwelling** |  |  |  |  |  |
| No | 17/58 (29.3) | 1 [Reference] | .12 | NA | NA |
| Yes | 89/431 (20.7) | 0.6 (0.3 – 1.2) |  | NA | NA |
| Missing Data | 5 (1.0) |  |  |  |  |
| **Frequency of information during the pandemic compared to before** |  |  |  |  |  |
| Less often | 18/67 (26.9) | 1 [Reference] | .17 | NA | NA |
| With the same frequency | 26/156 (16.7) | 0.5 (0.3 – 1.1) |  | NA | NA |
| More often | 61/264 (23.1) | 0.8 (0.4 – 1.5) |  | NA | NA |
| Missing Data | 7 (1.4) |  |  |  |  |
| **Feelings about the communication of COVID-19 pandemic medical data by the media** |  |  |  |  |  |
| Medium or total confidence | 44/250 (17.6) | 1 [Reference] | **.02** | NA | NA |
| Distrust or low confidence | 62/239 (25.9) | 1.7 (1.1 – 2.6) |  | NA | NA |
| Missing Data | 5 (1.0) |  |  |  |  |
| **Feelings on media communication about personal preventing measures** |  |  |  |  |  |
| Medium or total confidence | 67/338 (19.8) | 1 [Reference] | .13 | NA | NA |
| Distrust or low confidence | 39/149 (26.2) | 1.4 (0.9 – 2.2) |  | NA | NA |
| Missing Data | 7 (1.4) |  |  |  |  |
| **Feelings on media communication about the means to fight measures** |  |  |  |  |  |
| Medium or total confidence | 45/241 (18.7) | 1 [Reference] | .15 | NA | NA |
| Distrust or low confidence | 60/246 (24.4) | 1.4 (0.9 – 2.2) |  | NA | NA |
| Missing Data | 7 (1.4) |  |  |  |  |
| **Perception of information about the pandemic in the center** |  |  |  |  |  |
| Insufficient | 22/54 (40.7) | 1 [Reference] | **.0003** | NA | NA |
| Medium | 42/169 (24.8) | 0.5 (0.3 – 0.9) |  | NA | NA |
| Excellent | 42/264 (15.9) | 0.3 (0.1 – 0.5) |  | NA | NA |
| Missing Data | 7 (1.4) |  |  |  |  |
| **Perception of the level of personal protective equipment** |  |  |  |  |  |
| Insufficient | 29/65 (44.6) | 1 [Reference] | **<.0001** | NA | NA |
| Medium | 30/129 (23.3) | 0.4 (0.2 – 0.8) |  | NA | NA |
| Sufficient | 47/296 (15.9) | 0.2 (0.1 – 0.4) |  | NA | NA |
| Missing Data | 4 (0.8) |  |  |  |  |
| **Personal shortage** |  |  |  |  |  |
| Never/Rarely/Sometimes | 40/250 (16.0) | 1 [Reference] | **.003** | NA | NA |
| Frequently/Very frequently | 66/240 (27.5) | 1.9 (1.2 – 3.1) |  | NA | NA |
| Missing Data | 4 (0.8) |  |  |  |  |
| **Conflicts with residents relatives** |  |  |  |  |  |
| Never/Rarely/Sometimes | 61/343 (17.8) | 1 [Reference] | **.002** | NA | NA |
| Frequently/Very frequently | 45/146 (30.8) | 2.1 (1.3 – 3.3) |  | NA | NA |
| Missing Data | 5 (1.0) |  |  |  |  |
| **Conflicts with colleagues** |  |  |  |  |  |
| Never/Rarely/Sometimes | 81/448 (18.1) | 1 [Reference] | **<.0001** | 1 [Reference] | **.001** |
| Frequently/Very frequently | 23/40 (57.5) | 5.9 (3.0 – 11.8) |  | 3.9 (1.6 – 9.0) |  |
| Missing Data | 6 (1.2) |  |  |  |  |
| **Conflicts with residents** |  |  |  |  |  |
| Never/Rarely/Sometimes | 74/416 (17.8) | 1 [Reference] | **<.0001** | 1 [Reference] | **.008** |
| Frequently/Very frequently | 31/73 (42.5) | 3.4 (2.0 – 5.9) |  | 2.3 (1.2 – 4.4) |  |
| Missing Data | 5 (1.0) |  |  |  |  |
| **Accompanying COVID residents at the end-of-life** |  |  |  |  |  |
| No | 61/312 (19.6) | 1 [Reference] | .11 | NA | NA |
| Yes | 45/176 (25.6) | 1.5 (0.9 – 2.3) |  | NA | NA |
| Missing Data | 6 (1.2) |  |  |  |  |
| **People at risk for severe COVID-19** |  |  |  |  |  |
| No | 70/371 (18.9) | 1 [Reference] | **.01** | NA | NA |
| Yes | 36/119 (30.3) | 1.9 (1.2 – 3.0) |  | NA | NA |
| Missing Data | 4 (0.8) |  |  |  |  |
| **Reaction to holiday cancellation** |  |  |  |  |  |
| - Not concerned/It’s normal, I’m there for that, I’m useful to the residents/It’s part of the job, but it’s difficult, I’m tired | 74/408 (18.1) | 1 [Reference] | **<.0001** | 1 [Reference] | **.002** |
| - This is part of the job but I think that there are not enough of us and it is always the same people who work more | 11/47 (23.4) | 1.4 (0.7 – 2.8) |  | 0.9 (0.4 – 2.1) |  |
| - I’m so tired that I’m thinking of changing my assignment or job | 18/28 (64.3) | 8.1 (3.6 – 18.3) |  | 4.8 (2.0 – 11.7) |  |
| Missing Data | 11 (2.2) |  |  |  |  |
| **Nursing homes characteristics** |  |  |  |  |  |
| **Level of severity of the episode** |  |  |  |  |  |
| FREE-COVID/Unqualified episode | 74/312 (23.7) | 1 [Reference] | .13 | 1 [Reference] | **.03** |
| Severe episode/Critical episode | 32/178 (18.0) | 0.7 (0.4 – 1.1) |  | 0.5 (0.3 – 0.9) |  |
| Missing Data | 4 (0.8) |  |  |  |  |
| **Transfer of COVID residents to another institution** |  |  |  |  |  |
| No | 76/316 (24.1) | 1 [Reference] | .08 | NA | NA |
| Yes | 28/160 (17.5) | 0.6 (0.3 – 1.1) |  | NA | NA |
| Missing Data | 18 (3.6) |  |  |  |  |
| **Use of temporary workers before the pandemic** |  |  |  |  |  |
| No | 22/167 (13.2) | 1 [Reference] | **.001** | 1 [Reference] | **.002** |
| Sometimes | 52/218 (23.9) | 2.1 (1.2 – 3.6) |  | 1.6 (0.9 – 3.0) |  |
| Often | 32/98 (32.7) | 3.2 (1.7 – 5.9) |  | 3.4 (1.7 – 6.9) |  |
| Missing Data | 11 (2.2) |  |  |  |  |
| **^*^**According to the order of appearance of the survey forms, an imbalance in the completion rate was noted between the first questionnaire (PCL-5) and the last form (Socio-demographic questionnaire used to research the factors associated with the psychological disorders studied) (higher completion rate for the first questionnaire). In order to evaluate the prevalence associated with psychological disorders, all the answers filled in for each scale of evaluation of the latter were taken into account, although the questionnaire was not completed in full. For this reason, a difference in the numbers analyzed (between those for the prevalence of post-traumatic stress and those for the analysis of associated factors) is observed (see Figure 1). The search for factors associated with the occurrence of psychological disorders was carried out on 494 people (those who completed all the survey forms)  PTSD: Post-Traumatic Stress Disorder  ^ƚ^ No. /Total No.: Number of observations / Total number of observations  ^ǂ^ The results presented correspond to the pre-selection of variables at p value ≤ 20%. A second selection of variables was made at the 5% threshold and then integrated into the multivariate model  **^§^** AOR: Adjusted odd ratio with 95% confidence interval  **^**^** NA: Not Applicable | | | | | |

| **Table 2. Supplemental material. Pearson correlation coefficients between all scales** | | | | | | |
| --- | --- | --- | --- | --- | --- | --- |
|  | **PCL-5 score (95% CI)** | **HADS Anxiety score (95% CI)** | **HADS Depression score (95% CI)** | **MBI Emotional Exhaustion score (95% CI)** | **MBI Depersonalization score (95% CI)** | **MBI Personal Accomplishment score (95% CI)** |
| **PCL-5** | 1 | 0.72 (0.67 – 0.76) | 0.64 (0.59 – 0.69) | 0.62 (0.56 – 0.67) | 0.43 (0.35 – 0.50) | - 0.22 (- 0.31 – - 0.13) |
| **HADS Anxiety** |  | 1 | 0.60 (0.54 – 0.65) | 0.57 (0.51 – 0.63) | 0.39 (0.31 – 0.47) | - 0.28 (- 0.36 – - 0.19) |
| **HADS Depression** |  |  | 1 | 0.61 (0.55 – 0.66) | 0.38 (0.30 – 0.45) | - 0.34 (- 0.42 – - 0.26) |
| **MBI Emotional Exhaustion** |  |  |  | 1 | 0.60 (0.54 – 0.66) | - 0.26 (- 0.35 – - 0.18) |
| **MBI Depersonalization** |  |  |  |  | 1 | - 0.25 (- 0.33 – - 0.16) |
| **MBI Personal Accomplishment** |  |  |  |  |  | 1 |

PCL-5: PTSD Checklist for DSM-5; HADS: Hospital Anxiety and Depression Scale; MBI: Maslach Burnout Inventory

| **Table 3. Supplemental material. Factors associated with the anxiety gradient** | | | | | |
| --- | --- | --- | --- | --- | --- |
| **ANXIETY** | | | | | |
| **N = 494^*^** | **No^ƚ^. /Total No. (%)** | **Univariate analysis^ǂ^** | | **Multivariate analysis^*^ (N = 479)** | |
|  |  | **OR (95% CI)** | **p value** | **AOR (95% CI)^§^** | **p value** |
| **Staff characteristics** |  |  |  |  |  |
| **Fear for managing COVID-19 residents** |  |  |  |  |  |
| No/Somewhat | 62/290 (21.4) | 1 [Reference] | **<.0001** | 1 [Reference] | **<.0001** |
| Moderately | 31/94 (33.0) | 1.9 (1.2 – 3.0) |  | 1.8 (1.1 – 2.8) |  |
| A lot | 47/99 (47.5) | 3.2 (2.0 – 4.9) |  | 2.8 (1.7 – 4.4) |  |
| Missing Data | 11 (2.2) |  |  |  |  |
| **Age (years)** |  |  |  |  |  |
| < 30 and ≥ 50 | 61/245 (24.9) | 1 [Reference] | **.01** | 1 [Reference] | **.002** |
| 30 – 49 | 79/239 (33.1) | 1.5 (1.1 – 2.2) |  | 1.7 (1.2 – 2.5) |  |
| Missing Data | 10 (2.0) |  |  |  |  |
| **Personal shortage** |  |  |  |  |  |
| Never/Rarely/Sometimes | 57/249 (22.9) | 1 [Reference] | **.003** | NA**^**^** | NA |
| Frequently/Very frequently | 83/236 (35.2) | 1.7 (1.2 – 2.3) |  | NA | NA |
| Missing Data | 9 (1.8) |  |  |  |  |
| **Feelings about the communication of COVID-19 pandemic medical data by the media** |  |  |  |  |  |
| Medium or total confidence | 58/250 (23.2) | 1 [Reference] | **.0001** | 1 [Reference] | **.0002** |
| Distrust or low confidence | 80/233 (34.3) | 2.0 (1.4 – 2.8) |  | 2.0 (1.4 – 2.8) |  |
| Missing Data | 11 (2.2) |  |  |  |  |
| **Feelings on media communication about personal preventing measures** |  |  |  |  |  |
| Medium or total confidence | 85/335 (25.4) | 1 [Reference] | **.01** | NA | NA |
| Distrust or low confidence | 53/147 (36.1) | 1.6 (1.1 – 2.3) |  | NA | NA |
| Missing Data | 12 (2.4) |  |  |  |  |
| **Feelings on media communication about the means to fight measures** |  |  |  |  |  |
| Medium or total confidence | 56/240 (23.3) | 1 [Reference] | **.001** | NA | NA |
| Distrust or low confidence | 83/241 (34.4) | 1.7 (1.2 – 2.4) |  | NA | NA |
| Missing Data | 13 (2.6) |  |  |  |  |
| **Perception of information about the pandemic in the center** |  |  |  |  |  |
| Insufficient | 20/52 (38.5) | 1 [Reference] | **.03** | NA | NA |
| Medium | 49/167 (29.3) | 0.7 (0.4 – 1.2) |  | NA | NA |
| Excellent | 70/264 (26.5) | 0.5 (0.3 – 0.9) |  | NA | NA |
| Missing Data | 11 (2.2) |  |  |  |  |
| **Perception of the level of personal protective equipment** |  |  |  |  |  |
| Insufficient | 27/63 (42.9) | 1 [Reference] | **.009** | NA | NA |
| Medium | 39/128 (30.5) | 0.6 (0.3 – 1.1) |  | NA | NA |
| Sufficient | 74/294 (25.2) | 0.5 (0.3 – 0.8) |  | NA | NA |
| Missing Data | 9 (1.8) |  |  |  |  |
| **Same family environment as before the pandemic** |  |  |  |  |  |
| No | 12/32 (37.5) | 1 [Reference] | **.04** | 1 [Reference] | **.009** |
| Yes | 128/453 (28.3) | 0.5 (0.2 – 0.9) |  | 0.4 (0.2 – 0.8) |  |
| Missing Data | 9 (1.8) |  |  |  |  |
| **Conflicts with colleagues** |  |  |  |  |  |
| Never/Rarely/Sometimes | 121/444 (27.3) | 1 [Reference] | **.0009** | NA | NA |
| Frequently/Very frequently | 17/39 (43.6) | 2.9 (1.6 – 5.5) |  | NA | NA |
| Missing Data | 11 (2.2) |  |  |  |  |
| **Conflicts with residents** |  |  |  |  |  |
| Never/Rarely/Sometimes | 102/412 (24.8) | 1 [Reference] | **<.0001** | 1 [Reference] | **<.0001** |
| Frequently/Very frequently | 37/72 (51.4) | 3.3 (2.0 – 5.4) |  | 2.9 (1.8 – 4.9) |  |
| Missing Data | 10 (2.0) |  |  |  |  |
| **Nursing homes characteristics** |  |  |  |  |  |
| **Level of severity of the episode** |  |  |  |  |  |
| FREE-COVID/Unqualified episode | 88/312 (28.2) | 1 [Reference] | .77 | 1 [Reference] | .84 |
| Severe episode/Critical episode | 52/173 (30.1) | 1.1 (0.7 – 1.6) |  | 1.0 (0.6 – 1.5) |  |
| Missing Data | 9 (1.8) |  |  |  |  |
| **^*^**According to the order of appearance of the survey forms, an imbalance in the completion rate was noted between the first questionnaire (PCL-5) and the last form (Socio-demographic questionnaire used to research the factors associated with the psychological disorders studied) (higher completion rate for the first questionnaire). In order to evaluate the prevalence associated with psychological disorders, all the answers filled in for each scale of evaluation of the latter were taken into account, although the questionnaire was not completed in full. For this reason, a difference in the numbers analyzed (between those for the prevalence of anxiety and those for the analysis of associated factors) is observed (see Figure 1). The search for factors associated with the occurrence of psychological disorders was carried out on 494 people (those who completed all the survey forms)  ^ƚ^ No. /Total No.: Number of observations / Total number of observations  ^ǂ^ The results presented correspond to the pre-selection of variables at p value ≤ 20%. A second selection of variables was made at the 5% threshold and then integrated into the multivariate model  **^§^** AOR: Adjusted odd ratio with 95% confidence interval  **^**^** NA: Not Applicable | | | | | |

| **Table 4. Supplemental material. Factors associated with the depression gradient** | | | | | |
| --- | --- | --- | --- | --- | --- |
| **DEPRESSION** | | | | | |
| **N = 494^*^** | **No** ^ƚ^**. /Total No. (%)** | **Univariate analysis**^ǂ^ | | **Multivariate analysis (N = 476)** | |
|  |  | **OR (95% CI)** | **p value** | **AOR (95% CI)^§^** | **p value** |
| **Staff characteristics** |  |  |  |  |  |
| **Fear for managing COVID-19 residents** |  |  |  |  |  |
| No/Somewhat | 22/292 (7.5) | 1 [Reference] | **.0003** | NA**^**^** | NA |
| Moderately | 11/96 (11.5) | 1.6 (0.9 – 2.6) |  | NA | NA |
| A lot | 17/100 (17.0) | 2.7 (1.7 – 4.3) |  | NA | NA |
| Missing Data | 6 (1.2) |  |  |  |  |
| **People at risk of developing a severe form of COVID-19** |  |  |  |  |  |
| No | 32/370 (75.5) | 1 [Reference] | **.001** | 1 [Reference] | **.003** |
| Yes | 18/120 (15.0) | 2.0 (1.3 – 3.1) |  | 2.0 (1.3 – 3.2) |  |
| Missing Data | 4 (0.8) |  |  |  |  |
| **Feelings about the communication of COVID-19 pandemic medical data by the media** |  |  |  |  |  |
| Medium or total confidence | 19/251 (7.6) | 1 [Reference] | **.002** | NA | NA |
| Distrust or low confidence | 30/237 (12.7) | 1.9 (1.3 – 2.8) |  | NA | NA |
| Missing Data | 6 (1.2) |  |  |  |  |
| **Feelings on media communication about personal preventing measures** |  |  |  |  |  |
| Medium or total confidence | 31/337 (9.2) | 1 [Reference] | **.006** | 1 [Reference] | **.01** |
| Distrust or low confidence | 19/150 (12.7) | 1.8 (1.2 – 2.7) |  | 1.8 (1.1 – 2.7) |  |
| Missing Data | 7 (1.4) |  |  |  |  |
| **Feelings on media communication about the means to fight measures** |  |  |  |  |  |
| Medium or total confidence | 19/241 (7.9) | 1 [Reference] | **.004** | NA | NA |
| Distrust or low confidence | 30/245 (12.2) | 1.8 (1.2 – 2.6) |  | NA | NA |
| Missing Data | 8 (1.6) |  |  |  |  |
| **Conflicts with residents relatives** |  |  |  |  |  |
| Never/Rarely/Sometimes | 30/343 (8.8) | 1 [Reference] | **.01** | NA | NA |
| Frequently/Very frequently | 20/146 (13.7) | 1.7 (1.1 – 2.6) |  | NA | NA |
| Missing Data | 5 (1.0) |  |  |  |  |
| **Conflicts with colleagues** |  |  |  |  |  |
| Never/Rarely/Sometimes | 34/449 (7.6) | 1 [Reference] | **<.0001** | 1 [Reference] | **.02** |
| Frequently/Very frequently | 15/39 (38.5) | 4.0 (2.1 – 7.7) |  | 2.3 (1.1 – 4.7) |  |
| Missing Data | 6 (1.2) |  |  |  |  |
| **Conflicts with residents** |  |  |  |  |  |
| Never/Rarely/Sometimes | 37/417 (8.9) | 1 [Reference] | **.0002** | 1 [Reference] | **.003** |
| Frequently/Very frequently | 13/72 (18.1) | 2.7 (1.6 – 4.5) |  | 2.3 (1.3 – 4.0) |  |
| Missing Data | 5 (1.0) |  |  |  |  |
| **Reaction to vacation cancellation** |  |  |  |  |  |
| - Not concerned/It’s normal, I’m there for that, I’m useful to the residents/It’s part of the job, but it’s difficult, I’m tired | 34/408 (8.3) | 1 [Reference] | **<.0001** | 1 [Reference] | **.001** |
| - This is part of the job but I think that there are not enough of us and it is always the same people who work more | 4/47 (8.5) | 1.2 (0.6 – 2.4) |  | 1.0 (0.5 – 2.1) |  |
| - I’m so tired that I’m thinking of changing my assignment or job | 12/29 (41.4) | 5.6 (2.7 – 11.8) |  | 4.1 (1.9 – 8.8) |  |
| Missing Data | 10 (2.0) |  |  |  |  |
| **Perception of information about the pandemic in the center** |  |  |  |  |  |
| Insufficient | 16/54 (29.6) | 1 [Reference] | **<.0001** | 1 [Reference] | **.01** |
| Medium | 12/169 (7.1) | 0.3 (0.2 – 0.6) |  | 0.5 (0.3 – 1.0) |  |
| Excellent | 22/264 (8.3) | 0.3 (0.1 – 0.5) |  | 0.4 (0.2 – 0.7) |  |
| Missing Data | 7 (1.4) |  |  |  |  |
| **Nursing homes characteristics** |  |  |  |  |  |
| **Level of severity of the episode** |  |  |  |  |  |
| FREE-COVID/Unqualified episode | 38/315 (12.1) | 1 [Reference] | .18 | 1 [Reference] | .16 |
| Severe episode/Critical episode | 12/175 (6.9) | 0.7 (0.4 – 1.2) |  | 0.7 (0.4 – 1.2) |  |
| Missing Data | 4 (0.8) |  |  |  |  |
| **Transfer of COVID residents to another institution** |  |  |  |  |  |
| No | 43/319 (13.5) | 1 [Reference] | .06 | NA | NA |
| Yes | 7/157 (4.5) | 0.6 (0.4 – 1.0) |  | NA | NA |
| Missing Data | 18 (3.7) |  |  |  |  |
| **^*^**According to the order of appearance of the survey forms, an imbalance in the completion rate was noted between the first questionnaire (PCL-5) and the last form (Socio-demographic questionnaire used to research the factors associated with the psychological disorders studied) (higher completion rate for the first questionnaire). In order to evaluate the prevalence associated with psychological disorders, all the answers filled in for each scale of evaluation of the latter were taken into account, although the questionnaire was not completed in full. For this reason, a difference in the numbers analyzed (between those for the prevalence of depression and those for the analysis of associated factors) is observed (see Figure 1). The search for factors associated with the occurrence of psychological disorders was carried out on 494 people (those who completed all the survey forms)  ^ƚ^ No. /Total No.: Number of observations / Total number of observations  ^ǂ^ The results presented correspond to the pre-selection of variables at p value ≤ 20%. A second selection of variables was made at the 5% threshold and then integrated into the multivariate model  **^§^** AOR: Adjusted odd ratio with 95% confidence interval  **^**^** NA: Not Applicable | | | | | |

| **Table 5. Supplemental material. Factors associated with the emotional exhaustion, depersonalization, and personal accomplishment scores** | | | | | | |
| --- | --- | --- | --- | --- | --- | --- |
| **EMOTIONAL EXHAUSTION** | | | | | | |
| **N = 494^*^** | **Score mean (SD)^ƚƚ^** | **Median (IQR)^ǂǂ^** | **Univariate analysis^ǂ^** | | **Multivariate analysis^*^ (N = 468)** | |
|  |  |  | ***β*^§§^** | **p value** | ***β*^§§^** | **p value** |
| **Staff characteristics** |  | |  |  |  |  |
| **Sex** |  | |  |  |  |  |
| Male (n = 50) | 22.7 (14.9) | 20.5 (26.0) | 1 [Reference] | .18 | NA**^**^** | NA |
| Female (n = 428) | 25.5 (14.3) | 25.0 (24.0) | 2.8 |  | NA | NA |
| Missing Data (n = 16) |  |  |  |  |  |  |
| **Age (years)** |  |  |  |  |  |  |
| < 30 and ≥ 50 (n = 245) | 24.4 (14.3) | 23.0 (22.0) | 1 [Reference] | **.06** | NA | NA |
| 30 – 49 (n = 234) | 26.2 (14.3) | 26.0 (25.0) | 2.5 |  | NA | NA |
| Missing Data (n = 15) |  |  |  |  |  |  |
| **Seniority in the institution (years)** |  |  |  |  |  |  |
| < 1 (n = 58) | 21.1 (13.8) | 17.0 (21.0) | 1 [Reference] | **.05** | NA | NA |
| 1 – 5 (n = 189) | 26.5 (14.5) | 26.0 (23.0) | 5.7 |  | NA | NA |
| 6 – 10 (n = 84) | 26.0 (14.1) | 26.5 (26.0) | 5.1 |  | NA | NA |
| > 10 (n = 147) | 24.8 (14.2) | 24.0 (24.0) | 3.9 |  | NA | NA |
| Missing Data (n = 16) |  |  |  |  |  |  |
| **Fear for managing COVID-19 residents** |  |  |  |  |  |  |
| No/Somewhat (n = 286) | 22.8 (14.0) | 21.5 (23.0) | 1 [Reference] | **<.0001** | 1 [Reference] | **.01** |
| Moderately (n = 95) | 27.9 (14.3) | 28.0 (22.0) | 5.1 |  | 4.2 |  |
| A lot (n = 97) | 29.8 (14.0) | 30.0 (24.0) | 6.8 |  | 2.6 |  |
| Missing Data (n = 16) |  |  |  |  |  |  |
| **Number of COVID tests performed** |  |  |  |  |  |  |
| Never / < 5 times (n = 74) | 21.9 (14.8) | 18.5 (24.0) | 1 [Reference] | **.07** | NA | NA |
| Between 5 and 10 times (n =128) | 26.2 (14.7) | 26.0 (24.5) | 4.2 |  | NA | NA |
| > 10 times (n = 278) | 25.7 (13.9) | 25.0 (23.0) | 4.4 |  | NA | NA |
| Missing Data (n = 14) |  |  |  |  |  |  |
| **Conflicts with colleagues** |  |  |  |  |  |  |
| Never/Rarely/Sometimes (n = 440) | 24.1 (13.8) | 23.0 (23.0) | 1 [Reference] | **<.0001** | 1 [Reference] | **.001** |
| Frequently/Very frequently (n = 39) | 38.2 (13.3) | 42.0 (13.0) | 13.8 |  | 7.4 |  |
| Missing Data (n = 15) |  |  |  |  |  |  |
| **Conflicts with residents** |  |  |  |  |  |  |
| Never/Rarely/Sometimes (n = 408) | 23.8 (14.1) | 22.0 (24.0) | 1 [Reference] | **<.0001** | 1 [Reference] | **.0001** |
| Frequently/Very frequently (n = 72) | 33.8 (12.6) | 35.0 (17.0) | 10.5 |  | 6.5 |  |
| Missing Data (n = 14) |  |  |  |  |  |  |
| **Conflicts with residents relatives** |  |  |  |  |  |  |
| Never/Rarely/Sometimes (n = 335) | 23.5 (13.9) | 22.0 (24.0) | 1 [Reference] | **<.0001** | NA | NA |
| Frequently/Very frequently (n = 144) | 29.5 (14.4) | 30.5 (23.0) | 6.4 |  | NA | NA |
| Missing Data (n = 15) |  |  |  |  |  |  |
| **Personal shortage** |  |  |  |  |  |  |
| Never/Rarely/Sometimes (n = 244) | 21.9 (13.4) | 20.0 (22.5) | 1 [Reference] | **<.0001** | 1 [Reference] | **.007** |
| Frequently/Very frequently (n = 236) | 28.7 (14.4) | 29.0 (24.0) | 6.5 |  | 3.3 |  |
| Missing Data (n = 14) |  |  |  |  |  |  |
| **Feelings about the communication of medical data on the pandemic by the media** |  |  |  |  |  |  |
| Medium or total confidence (n = 246) | 22.6 ( 14.0) | 20.0 (22.0) | 1 [Reference] | **<.0001** | 1 [Reference] | **.01** |
| Distrust or low confidence (n = 234) | 28.1 (14.1) | 29.5 (22.0) | 5.5 |  | 2.9 |  |
| Missing Data (n = 14) |  |  |  |  |  |  |
| **Feelings on media communication about personal preventing measures** |  |  |  |  |  |  |
| Medium or total confidence (n = 331) | 24.0 (14.0) | 22.0 (23.0) | 1 [Reference] | **.004** | NA | NA |
| Distrust or low confidence (n = 148) | 28.1 (14.8) | 29.5 (27.0) | 4.0 |  | NA | NA |
| Missing Data (n = 15) |  |  |  |  |  |  |
| **Feelings on media communication about the means to fight measures** |  |  |  |  |  |  |
| Medium or total confidence (n = 237) | 22.8 (14.6) | 20.0 (23.0) | 1 [Reference] | **.0002** | NA | NA |
| Distrust or low confidence (n = 240) | 27.8 (13.7) | 28.0 (21.0) | 4.7 |  | NA | NA |
| Missing Data (n = 17) |  |  |  |  |  |  |
| **Accompanying COVID residents at the end-of-life** |  |  |  |  |  |  |
| No (n = 307) | 24.1 (13.8) | 23.0 (22.0) | 1 [Reference] | **.01** | NA | NA |
| Yes (n = 171) | 27.4 (15.1) | 28.0 (27.0) | 3.6 |  | NA | NA |
| Missing Data (n = 16) |  |  |  |  |  |  |
| **Perception of information about the pandemic in the center** |  |  |  |  |  |  |
| Insufficient (n = 52) | 34.6 (13.8) | 37.5 (18.5) | 1 [Reference] | **<.0001** | 1 [Reference] | **.001** |
| Medium (n = 166) | 26.7 (13.9) | 27.0 (23.0) | - 11.9 |  | - 4.6 |  |
| Excellent (n = 259) | 22.6 (13.8) | 21.0 (23.0) | - 7.7 |  | - 7.2 |  |
| Missing Data (n = 17) |  |  |  |  |  |  |
| **Perception of the level of personal protective equipment** |  |  |  |  |  |  |
| Insufficient (n = 62) | 33.8 (13.8) | 36.5 (19.0) | 1 [Reference] | **<.0001** | NA | NA |
| Medium (n = 129) | 26.5 (14.6) | 26.0 (25.0) | - 7.0 |  | NA | NA |
| Sufficient (n = 289) | 22.9 (13.6) | 21.0 (21.0) | - 10.9 |  | NA | NA |
| Missing Data (n = 14) |  |  |  |  |  |  |
| **Reaction to holiday cancellation** |  |  |  |  |  |  |
| - Not concerned/It’s normal, I’m there for that, I’m useful to the residents/It’s part of the job, but it’s difficult, I’m tired (n = 399) | 23.6 (14.1) | 22.0 (23.0) | 1 [Reference] | **<.0001** | 1 [Reference] | **<.0001** |
| - This is part of the job but I think that there are not enough of us and it is always the same people who work more (n = 48) | 29.9 (12.0) | 33.0 (19.5) | 6.0 |  | 3.2 |  |
| - I’m so tired that I’m thinking of changing my assignment or job (n = 27) | 41.6 (8.5) | 43.0 (12.0) | 18.0 |  | 12.8 |  |
| Missing Data (n = 20) |  |  |  |  |  |  |
| **Nursing homes characteristics** |  |  |  |  |  |  |
| **Level of severity of the episode** |  |  |  |  |  |  |
| FREE-COVID/Unqualified episode (n = 307) | 25.5 (14.4) | 24.0 (24.0) | 1 [Reference] | .77 | 1 [Reference] | .35 |
| Severe episode/Critical episode (n = 173) | 24.8 (14.1) | 25.0 (23.0) | - 0.5 |  | - 1.4 |  |
| Missing Data (n =14) |  |  |  |  |  |  |
| **DEPERSONALIZATION** | | | | | | |
| **N = 494** | **Score mean (SD)** | **Median (IQR)** | **Univariate analysis** | | **Multivariate analysis (N = 459)** | |
|  |  |  | ***β*** | **p value** | ***β*** | **p value** |
| **Staff characteristics** |  |  |  |  |  |  |
| **Fear for managing COVID-19 residents** |  |  |  |  |  |  |
| No/Somewhat (n = 287) | 6.8 (6.3) | 5.0 (9.0) | 1 [Reference] | **.01** | 1 [Reference] | **.006** |
| Moderately (n = 94) | 9.1 (6.9) | 8.0 (12.0) | 2.2 |  | - 1.3 |  |
| A lot (n = 101) | 7.2 (6.6) | 6.0 (7.0) | 0.3 |  | 1.4 |  |
| Missing Data (n = 12) |  |  |  |  |  |  |
| **Personal shortage** |  |  |  |  |  |  |
| Never/Rarely/Sometimes (n = 245) | 6.3 (5.6) | 5.0 (7.0) | 1 [Reference] | **.0007** | NA | NA |
| Frequently/Very frequently (n = 238) | 8.4 (7.2) | 6.0 (10.0) | 2.0 |  | NA | NA |
| Missing Data (n = 11) |  |  |  |  |  |  |
| **Conflicts with colleagues** |  |  |  |  |  |  |
| Never/Rarely/Sometimes (n = 441) | 7.0 (6.3) | 6.0 (9.0) | 1 [Reference] | **.0002** | NA | NA |
| Frequently/Very frequently (n = 40) | 11.2 (7.6) | 10.5 (9.5) | 4.0 |  | NA | NA |
| Missing Data (n = 13) |  |  |  |  |  |  |
| **Conflicts with residents** |  |  |  |  |  |  |
| Never/Rarely/Sometimes (n = 409) | 6.5 (6.0) | 5.0 (8.0) | 1 [Reference] | **<.0001** | 1 [Reference] | **.001** |
| Frequently/Very frequently (n = 73) | 12.1 (7.3) | 12.0 (10.0) | 5.7 |  | 3.9 |  |
| Missing Data (n = 12) |  |  |  |  |  |  |
| **Conflicts with residents relatives** |  |  |  |  |  |  |
| Never/Rarely/Sometimes (n = 337) | 6.3 (5.9) | 5.0 (8.0) | 1 [Reference] | **<.0001** | 1 [Reference] | **.003** |
| Frequently/Very frequently (n = 145) | 9.7 (7.1) | 8.0 (11.0) | 3.4 |  | 1.9 |  |
| Missing Data (n = 12) |  |  |  |  |  |  |
| **Feelings about the communication of medical data on the pandemic by the media** |  |  |  |  |  |  |
| Medium or total confidence (n = 246) | 6.2 (6.0) | 4.5 (8.0) | 1 [Reference] | **<.0001** | NA | NA |
| Distrust or low confidence (n = 236) | 8.5 (6.8) | 7.0 (10.0) | 2.3 |  | NA | NA |
| Missing Data (n = 12) |  |  |  |  |  |  |
| **Feelings on media communication about personal preventing measures** |  |  |  |  |  |  |
| Medium or total confidence (n = 332) | 6.5 (6.1) | 5.0 (8.0) | 1 [Reference] | **<.0001** | 1 [Reference] | **.001** |
| Distrust or low confidence (n = 149) | 9.2 (7.1) | 8.0 (11.0) | 2.6 |  | 1.9 |  |
| Missing Data (n = 13) |  |  |  |  |  |  |
| **Feelings on media communication about the means to fight measures** |  |  |  |  |  |  |
| Medium or total confidence (n = 237) | 6.4 (6.3) | 4.0 (8.0) | 1 [Reference] | **.001** | NA | NA |
| Distrust or low confidence (n = 242) | 8.3 (6.6) | 7.0 (9.0) | 1.9 |  | NA | NA |
| Missing Data (n = 15) |  |  |  |  |  |  |
| **Number of COVID tests performed** |  |  |  |  |  |  |
| Never / < 5 times (n = 74) | 5.8 (6.0) | 4.5 (7.0) | 1 [Reference] | .12 | NA | NA |
| Between 5 and 10 times (n = 127) | 7.8 (6.9) | 6.0 (11.0) | 1.9 |  | NA | NA |
| > 10 times (n = 282) | 7.5 (6.4) | 6.0 (10.0) | 1.7 |  | NA | NA |
| Missing Data (n = 11) |  |  |  |  |  |  |
| **People at risk of developing a severe form of COVID-19** |  |  |  |  |  |  |
| No (n = 365) | 7.0 (6.4) | 6.0 (9.0) | 1 [Reference] | .09 | NA | NA |
| Yes (n = 118) | 8.3 (6.8) | 7.0 (11.0) | 1.2 |  | NA | NA |
| Missing Data (n = 11) |  |  |  |  |  |  |
| **Perception of the level of personal protective equipment** |  |  |  |  |  |  |
| Insufficient (n = 63) | 11.3 (7.3) | 12.0 (11.0) | 1 [Reference] | **<.0001** | 1 [Reference] | **.0002** |
| Medium (n = 130) | 8.0 (6.3) | 6.0 (9.0) | - 3.1 |  | - 1.8 |  |
| Sufficient (n = 290) | 6.2 (6.0) | 5.0 (8.0) | - 5.1 |  | - 3.4 |  |
| Missing Data (n = 11) |  |  |  |  |  |  |
| **Perception of information about the pandemic in the center** |  |  |  |  |  |  |
| Insufficient (n = 54) | 10.0 (6.6) | 9.5 (10.0) | 1 [Reference] | **.0004** | NA | NA |
| Medium (n = 165) | 7.9 (6.9) | 6.0 (10.0) | - 2.1 |  | NA | NA |
| Excellent (n = 261) | 6.4 (6.0) | 5.0 (7.0) | - 3.6 |  | NA | NA |
| Missing Data (n = 14) |  |  |  |  |  |  |
| **Reaction to holiday cancellation** |  |  |  |  |  |  |
| - Not concerned/It’s normal, I’m there for that, I’m useful to the residents/It’s part of the job, but it’s difficult, I’m tired (n = 401) | 6.7 (6.2) | 5.0 (8.0) | 1 [Reference] | **<.0001** | 1 [Reference] | **.007** |
| - This is part of the job but I think that there are not enough of us and it is always the same people who work more (n = 48) | 9.6 (7.1) | 9.0 (11.0) | 2.9 |  | 3.2 |  |
| - I’m so tired that I’m thinking of changing my assignment or job (n = 29) | 11.0 (5.9) | 11.0 (9.0) | 4.3 |  | 12.8 |  |
| Missing Data (n = 16) |  |  |  |  |  |  |
| **Nursing homes characteristics** |  |  |  |  |  |  |
| **Level of severity of the episode** | 6.1 (5.8) | 5.0 (7.5) |  |  |  |  |
| FREE-COVID/Unqualified episode (n = 311) | 7.4 (6.4) | 6.0 (10.0) | 1 [Reference] | .64 | 1 [Reference] | .62 |
| Severe episode/Critical episode (n = 172) | 7.2 (6.6) | 5.0 (9.0) | - 0.4 |  | - 0.4 |  |
| Missing Data (n = 11) |  |  |  |  |  |  |
| **Use of temporary workers during the pandemic** |  |  |  |  |  |  |
| No temporary staff (n = 144) |  |  | 1 [Reference] | .10 | 1 [Reference] | **.02** |
| Less often (n = 33) | 7.1 (5.4) | 6.0 (8.0) | 1.0 |  | 0.6 |  |
| Also often (n = 79) | 7.9 (7.3) | 6.0 (10.0) | 2.0 |  | 1.4 |  |
| More often (n = 213) | 8.2 (6.7) | 7.0 (10.0) | 2.0 |  | 2.2 |  |
| Missing Data (n = 25) |  |  |  |  |  |  |
| **Transfer of COVID residents to another institution** |  |  |  |  |  |  |
| No (n = 314) | 7.9 (6.5) | 6.0 (10.0) | 1 [Reference] | **.03** | 1 [Reference] | **.02** |
| Yes (n = 155) | 6.5 (6.4) | 5.0 (8.0) | - 1.7 |  | - 1.6 |  |
| Missing Data (n = 25) |  |  |  |  |  |  |
| **PERSONAL ACCOMPLISHMENT** | | | | | | |
| **N = 494** | **Score mean (SD)** | **Median (IQR)** | **Univariate analysis** | | **Multivariate analysis (N = 436)** | |
|  |  |  | ***β*** | **p value** | ***β*** | **p value** |
| **Staff characteristics** |  |  |  |  |  |  |
| **Fear for managing COVID-19 residents** |  |  |  |  |  |  |
| No/Somewhat (n = 279) | 38.7 (6.8) | 40.0 (8.0) | 1 [Reference] | **.001** | 1 [Reference] | **.001** |
| Moderately (n = 92) | 37.2 (6.3) | 37.5 (9.0) | - 1.5 |  | - 1.5 |  |
| A lot (n = 96) | 35.7 (7.9) | 37.0 (10.0) | - 2.9 |  | - 2.8 |  |
| Missing Data (n = 27) |  |  |  |  |  |  |
| **Occupation** |  |  |  |  |  |  |
| Caregiver (n = 350) | 38.1 (7.0) | 39.0 (9.0) | 1 [Reference] | **.03** | 1 [Reference] | **.007** |
| Non-caregiver (n = 119) | 36.7 (7.2) | 37.0 (11.0) | - 1.6 |  | - 2.0 |  |
| Missing Data (n = 25) |  |  |  |  |  |  |
| **Conflicts with residents’ relatives** |  |  |  |  |  |  |
| Never/Rarely/Sometimes (n = 325) | 38.4 (6.8) | 40.0 (8.0) | 1 [Reference] | **.0008** | 1 [Reference] | **.006** |
| Frequently/Very frequently (n = 143) | 36.2 (7.4) | 37.0 (8.0) | - 2.3 |  | - 1.9 |  |
| Missing Data (n = 26) |  |  |  |  |  |  |
| **Conflicts with residents** |  |  |  |  |  |  |
| Never/Rarely/Sometimes (n = 399) | 38.1 (7.0) | 39.0 (9.0) | 1 [Reference] | **.05** | NA | NA |
| Frequently/Very frequently (n = 69) | 36.3 (6.7) | 37.0 (8.0) | - 1.8 |  | NA | NA |
| Missing Data (n = 26) |  |  |  |  |  |  |
| **People at risk of developing a severe form of COVID-19** |  |  |  |  |  |  |
| No (n = 355) | 38.1 (6.9) | 39.0 (9.0) | 1 [Reference] | **.16** | NA | NA |
| Yes (n = 114) | 36.9 (7.3) | 37.0 (10.0) | - 1.1 |  | NA | NA |
| Missing Data (n = 25) |  |  |  |  |  |  |
| **Same family environment as before the pandemic** |  |  |  |  |  |  |
| No (n = 31) | 35.6 (8.1) | 37.0 (10.0) | 1 [Reference] | **.08** | NA | NA |
| Yes (n = 438) | 37.9 (6.9) | 39.0 (9.0) | 2.3 |  | NA | NA |
| Missing Data (n = 25) |  |  |  |  |  |  |
| **Frequency of information during the pandemic compared to before** |  |  |  |  |  |  |
| Less often (n = 65) | 34.8 (9.6) | 37.0 (10.0) | 1 [Reference] | **.0006** | NA | NA |
| With the same frequency (n = 154) | 38.6 (6.1) | 39.0 (9.0) | 3.8 |  | NA | NA |
| More often (n = 248) | 38.1 (6.5) | 39.0 (8.5) | 3.4 |  | NA | NA |
| Missing Data (n = 27) |  |  |  |  |  |  |
| **Feelings about the communication of medical data on the pandemic by the media** |  |  |  |  |  |  |
| Medium or total confidence (n = 238) | 38.7 (6.8) | 40.0 (8.0) | 1 [Reference] | **.005** | NA |  |
| Distrust or low confidence (n = 230) | 36.8 (7.2) | 37.0 (9.0) | - 1.8 |  | NA | NA |
| Missing Data (n = 26) |  |  |  |  |  | NA |
| **Feelings on media communication about the means to fight measures** |  |  |  |  |  |  |
| Medium or total confidence (n = 227) | 38.4 (7.0) | 40.0 (9.0) | 1 [Reference] | .09 | NA | NA |
| Distrust or low confidence (n = 239) | 37.2 (7.0) | 38.0 (9.0) | - 1.1 |  | NA | NA |
| Missing Data (n = 28) |  |  |  |  |  |  |
| **Reaction to holiday cancellation** |  |  |  |  |  |  |
| - Not concerned/It’s normal, I’m there for that, I’m useful to the residents/It’s part of the job, but it’s difficult, I’m tired (n = 388) | 38.1 (6.9) | 39.0 (9.0) | 1 [Reference] | .15 | NA | NA |
| - This is part of the job but I think that there are not enough of us and it is always (n = 47)   the same people who work more | 36.2 (6.5) | 36.0 (9.0) | - 1.9 |  | NA | NA |
| - I’m so tired that I’m thinking of changing my assignment or job (n = 28) | 36.7 (7.9) | 37.5 (8.5) | - 1.3 |  | NA | NA |
| Missing Data (n = 31) |  |  |  |  |  |  |
| **Nursing homes characteristics** |  |  |  |  |  |  |
| **Level of severity of the episode** |  |  |  |  |  |  |
| FREE-COVID/Unqualified episode (n = 303) | 37.7 (6.6) | 38.0 (8.0) | 1 [Reference] | .97 | 1 [Reference] | .52 |
| Severe episode/Critical episode (n = 166) | 37.8 (7.7) | 39.5 (9.0) | 0.02 |  | 0.5 |  |
| Missing Data (n = 25) |  |  |  |  |  |  |
| **^*^**According to the order of appearance of the survey forms, an imbalance in the completion rate was noted between the first questionnaire (PCL-5) and the last form (Socio-demographic questionnaire used to research the factors associated with the psychological disorders studied) (higher completion rate for the first questionnaire). In order to evaluate the prevalence associated with psychological disorders, all the answers filled in for each scale of evaluation of the latter were taken into account, although the questionnaire was not completed in full. The search for factors associated with the emotional exhaustion, depersonalization, and personal accomplishment scores was carried out on 494 people (those who completed all the survey forms)  ^ǂ^ The results presented correspond to the pre-selection of variables at p value ≤ 20%. A second selection of variables was made at the 5% threshold and then integrated into the multivariate model  **^**^** NA: Not Applicable  ^ƚƚ^ SD : Standard deviation  **^ǂǂ^** IQR : Interquartile range  **^§§^** Regression coefficient | | | | | | |
