# Supplementary material for: Post-traumatic stress disorder, anxiety, depression and burnout in nursing home staff in South France during the COVID-19 pandemic
Source: Transl Psychiatry. 2023 Jun 15;13:205. doi: 10.1038/s41398-023-02488-1 (PMC10267542; doi:10.1038/s41398-023-02488-1)
Supplement: Supplementary file 2 — Supplemental material (Figures) [file 41398_2023_2488_MOESM2_ESM.pptx]

## Slide 1
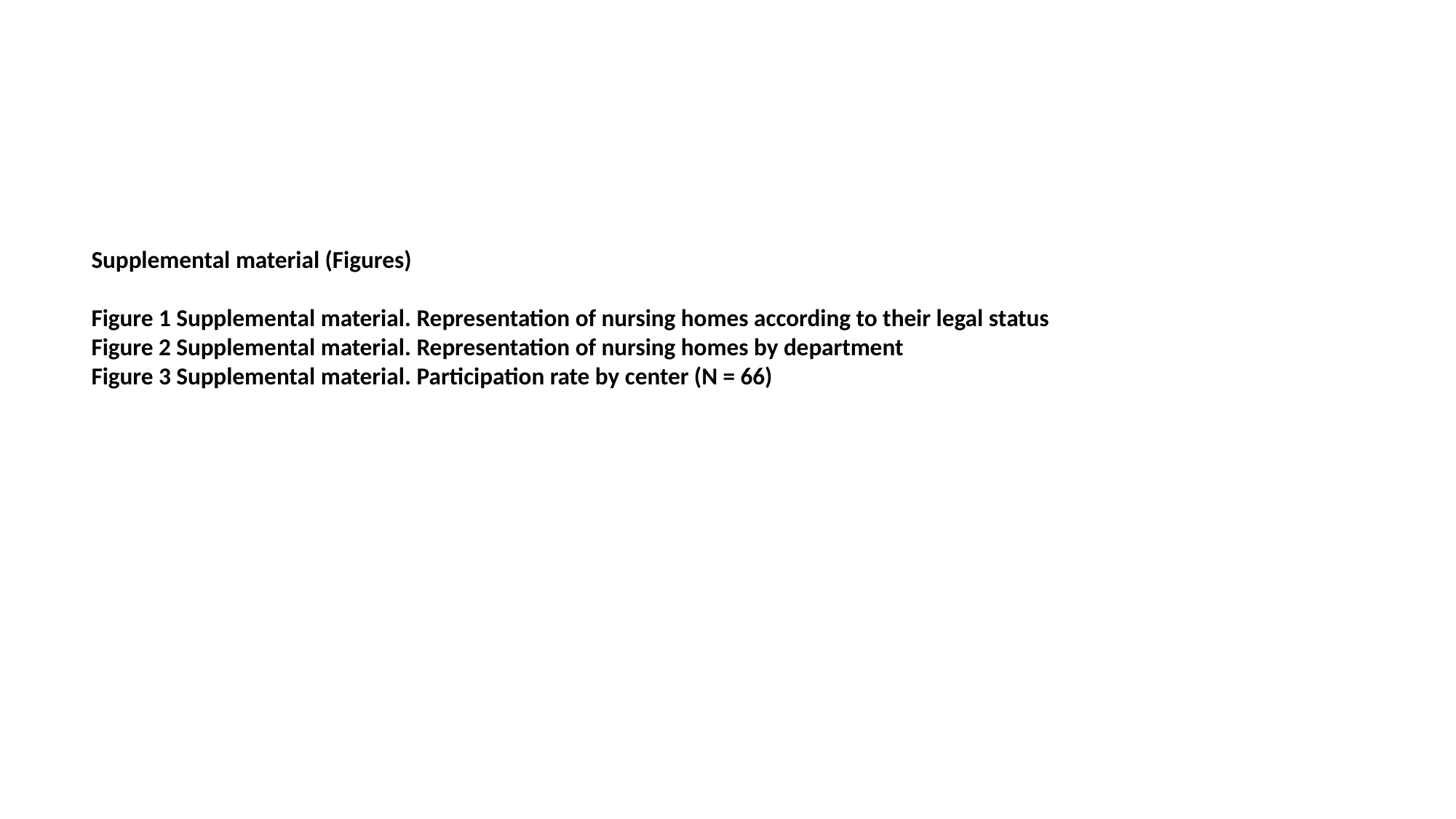

Supplemental material (Figures)
Figure 1 Supplemental material. Representation of nursing homes according to their legal status
Figure 2 Supplemental material. Representation of nursing homes by department
Figure 3 Supplemental material. Participation rate by center (N = 66)

## Slide 2
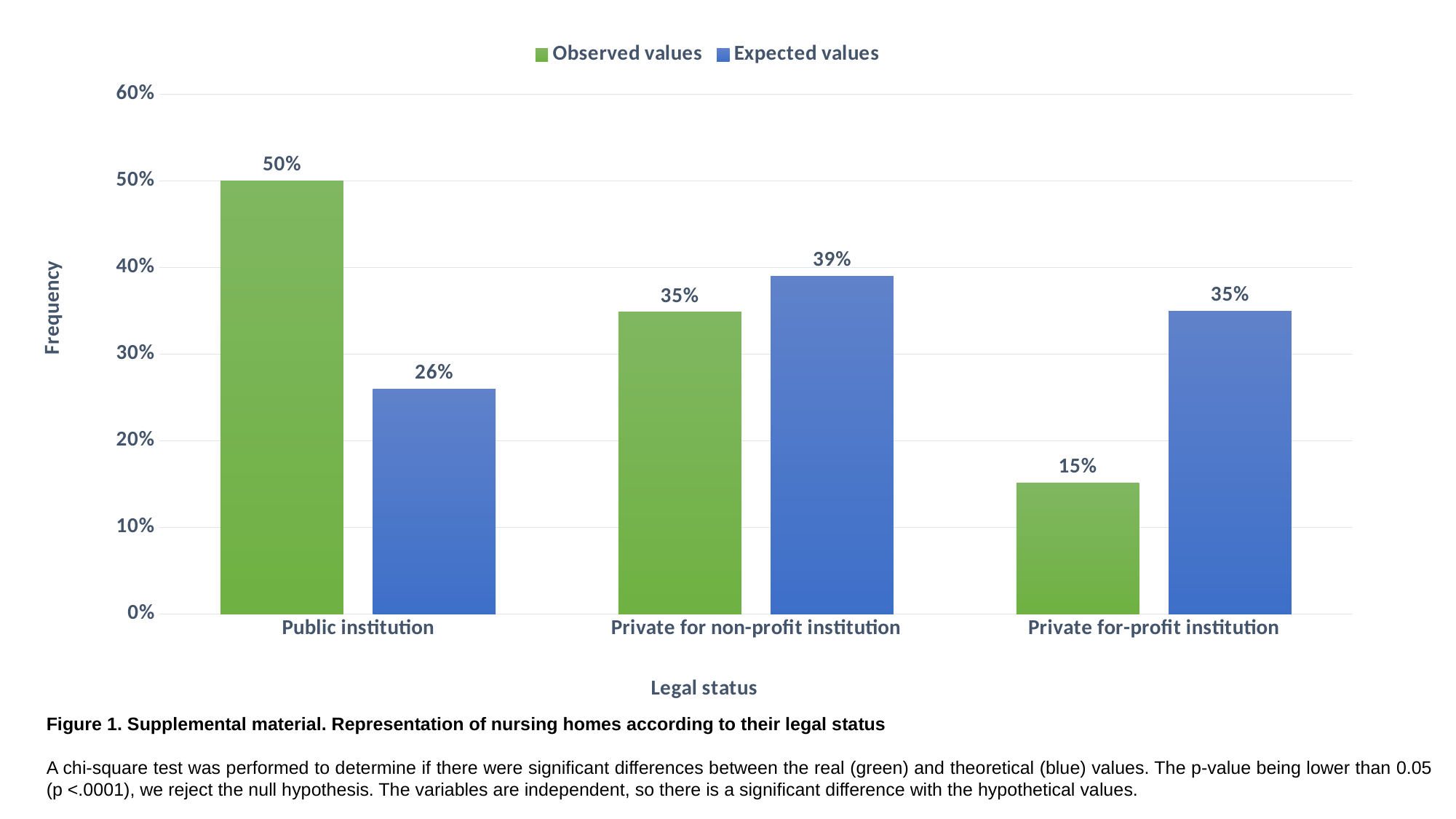

### Chart
| Category | Observed values | Expected values |
|---|---|---|
| Public institution | 0.5 | 0.26 |
| Private for non-profit institution | 0.3485 | 0.39 |
| Private for-profit institution | 0.1515 | 0.35 |Figure 1. Supplemental material. Representation of nursing homes according to their legal status
A chi-square test was performed to determine if there were significant differences between the real (green) and theoretical (blue) values. The p-value being lower than 0.05 (p <.0001), we reject the null hypothesis. The variables are independent, so there is a significant difference with the hypothetical values.

## Slide 3
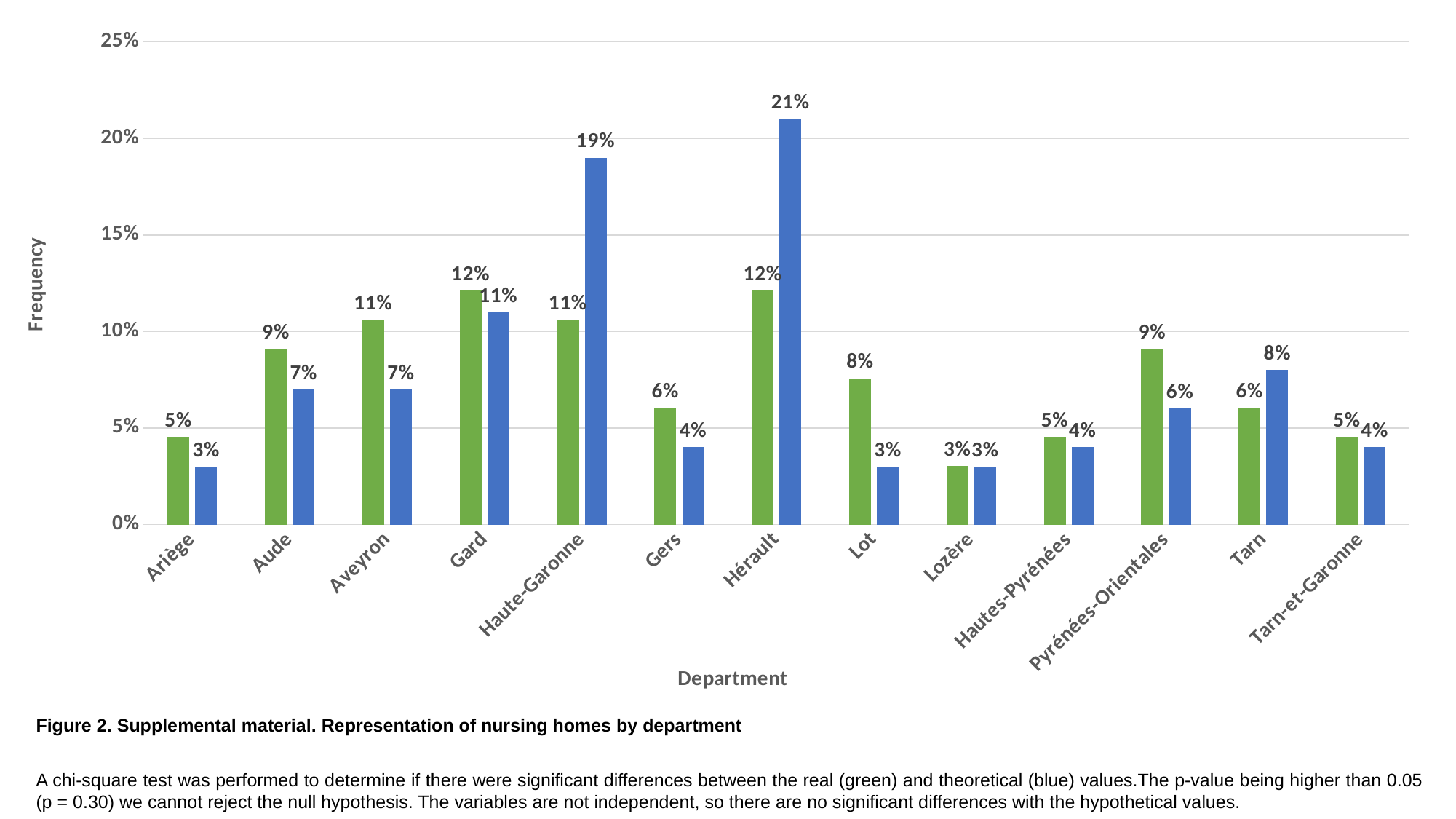

### Chart
| Category | Expected value | Observed value |
|---|---|---|
| Ariège | 0.0455 | 0.03 |
| Aude | 0.0909 | 0.07 |
| Aveyron | 0.1061 | 0.07 |
| Gard | 0.1212 | 0.11 |
| Haute-Garonne | 0.1061 | 0.19 |
| Gers | 0.0606 | 0.04 |
| Hérault | 0.1212 | 0.21 |
| Lot | 0.0758 | 0.03 |
| Lozère | 0.0303 | 0.03 |
| Hautes-Pyrénées | 0.0455 | 0.04 |
| Pyrénées-Orientales | 0.0909 | 0.06 |
| Tarn | 0.0606 | 0.08 |
| Tarn-et-Garonne | 0.0455 | 0.04 |Figure 2. Supplemental material. Representation of nursing homes by department
A chi-square test was performed to determine if there were significant differences between the real (green) and theoretical (blue) values.The p-value being higher than 0.05 (p = 0.30) we cannot reject the null hypothesis. The variables are not independent, so there are no significant differences with the hypothetical values.

## Slide 4
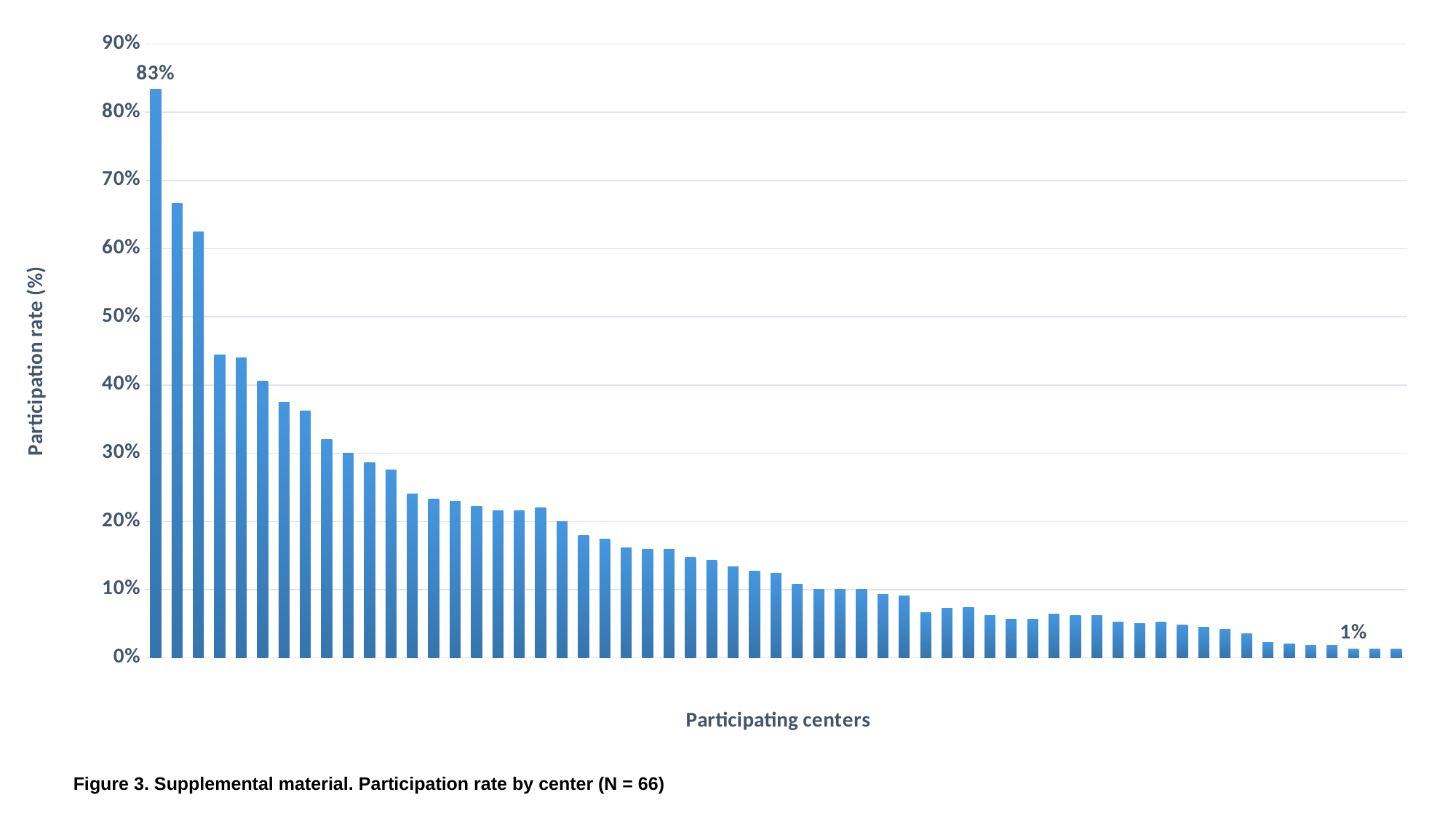

### Chart
| Category | Taux de participation (%)/centre |
|---|---|Figure 3. Supplemental material. Participation rate by center (N = 66)
